# Direct Formation of the Atomic Pd-ZnO Interface by Magnetron Sputtering Primed for Methanol Production from CO_2_

**DOI:** 10.1021/acscatal.5c04822

**Published:** 2025-08-22

**Authors:** Louise R. Smith, Emerson C. Kohlrausch, Kieran J. Aggett, Yifan Chen, Isla E. Gow, Andreas Weilhard, Luke T. Norman, Wolfgang Theis, David J. Morgan, Liam Bailey, Andrei N. Khlobystov, Jesum Alves Fernandes, Graham J. Hutchings

**Affiliations:** † Max Planck-Cardiff Centre on the Fundamentals of Heterogeneous Catalysis FUNCAT, Translational Research Hub, 2112 Cardiff University, Maindy Road, Cardiff CF24 4HQ, U.K.; ‡ School of Chemistry, 6123 University of Nottingham, University Park, Nottingham NG7 2RD, U.K.; § Nanoscale Physics Research Laboratory, School of Physics and Astronomy, 1724 University of Birmingham, Edgbaston B15 2TT, U.K.

**Keywords:** methanol, carbon dioxide, magnetron sputtering, palladium@zinc, heterogeneous catalysis

## Abstract

Carbon dioxide is not only a greenhouse gas but also a valuable feedstock for producing chemicals and fuels, especially methanol, which serves as an energy storage medium and a precursor for olefins and gasoline. Herein, we show that a clean, atomically defined interface between a Pd catalyst and a ZnO support allows for the direct production of methanol from CO_2_ without any catalyst activation or induction period. Using magnetron sputtering, Pd atoms are directly deposited onto the ZnO surface, self-assembling into Pd nanoclusters with a high fraction of surface atoms, driven solely by the surface chemistry of ZnO, eliminating the need for solvents, reagents, or ligands. This atomically defined Pd/ZnO interface facilitates Pd–Zn alloying in situ during the reaction, achieving an impressive methanol production rate of 16.4 mol h^–1^ mol^–1^_Pd_, outperforming catalysts prepared by other methods. By eliminating interfacial impurities and the consequent need for pretreatment, our work establishes magnetron sputtering as a transformative method for fabricating high-performance catalysts.

## Graphical abstract

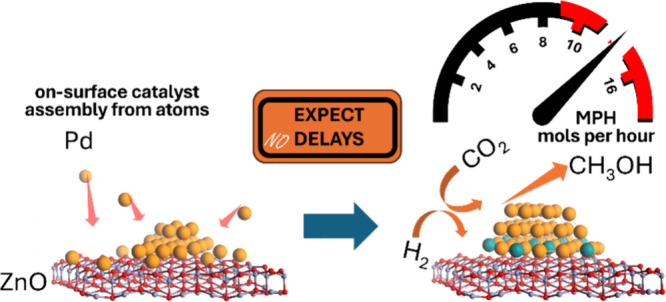

## Introduction

The direct hydrogenation of carbon dioxide to methanol (CO_2_ + 3H_2_ ⇄ CH_3_OH + H_2_O) is a crucial pathway for producing sustainable fuels and chemicals from CO_2_. The production of methanol from CO_2_ and H_2_ from renewable energy sources underpins the concept of the methanol economy, first proposed by Olah.^1^ In this framework, methanol is used as an energy storage vector, fuel, and feedstock for the synthesis of olefins and gasoline through the MTO (methanol to olefins) and MTG (methanol to gasoline) processes, respectively, thus reducing fossil fuel dependency and ultimately achieving net zero. Consequently, the development of catalysts for the hydrogenation of CO_2_ to methanol has been a major focus of research.

Pd/ZnO catalysts have received significant attention for CO_2_ hydrogenation, and the β-PdZn 1:1 alloy (denoted as PdZn) has been shown to be crucial in maximizing methanol production.^2–5^ Recent studies investigating the effect of Zn loading using PdZn/TiO_2_ catalysts showed that both the PdZn alloy and ZnO phase are required to achieve high methanol yields, with Quilis et al. highlighting the importance of PdZn/ZnO interfaces.^6,7^ van Bokhoven and co-workers used a range of in situ and operando techniques to study the reaction, proposing a bifunctional catalytic mechanism whereby CO_2_ activation occurs on the ZnO phase while hydrogen activation takes place on the PdZn alloy. This enables the hydrogenation of the formate intermediate to methanol at the PdZn/ZnO interface.^5^ Previous work has demonstrated that the PdZn alloy can be formed from Pd/ZnO catalysts through reductive pretreatment,^2^ or upon exposure to reaction conditions.^3^

Pd/ZnO catalysts prepared by sol immobilization showed a strong correlation between PdZn particle size and methanol production, with methanol selectivity decreasing from 60 to 20% upon increasing PdZn particle size from 3 to 7 nm, highlighting the importance of small PdZn nanoparticles with a narrow size distribution in maximizing methanol productivity.^2^ Chemical vapor impregnation (CVI) has been shown to be a highly efficient method for preparing PdZn alloy catalysts for CO_2_ hydrogenation. The high activity is attributed to the narrow particle size distribution, ready PdZn alloy formation, and high thermal stability under reducing atmospheres.^3,4,8,9^ CVI is a solventless catalyst preparation technique that involves combining volatile metal precursors (e.g., metal acetylacetonates) with the catalyst support, followed by heating under vacuum to induce precursor sublimation and deposition onto the support.^10,11^ Subsequent calcination treatments, which decompose and remove the ligands, are required to yield a high-performing supported metal catalyst for methanol production.^9^ Consequently, the primary challenge in catalyst development for methanol production from CO_2_ is to design a catalyst that rapidly forms a clean PdZn alloy, which enhances the interfacial charge transfer between these key elements, ultimately leading to a high methanol productivity. Additionally, the formation of the catalyst–support interface must be both energy-efficient and atom-efficient, requiring minimal activation and generating as little waste as possible.

In this work, we demonstrate that the direct deposition of Pd atoms onto ZnO surfaces enables rapid PdZn alloying, resulting in a catalyst for methanol synthesis from CO_2_ that outperforms those prepared from Pd compounds. We utilized magnetron sputtering (MS) to generate a flow of Pd atoms onto the ZnO support. The individual Pd atoms self-assemble into nanoclusters, guided exclusively by surface properties such as diffusion barriers and the density of surface defects, thereby controlling the nanocluster size distribution without requiring chemical reagents.^12–17^ Importantly, our approach eliminates the need to reduce cationic metal species or burn off organic ligands, enabling a direct formation clean atomic Pd/ZnO interface, which we demonstrate as key to the rapid formation of the PdZn alloy—crucial for achieving higher methanol yield and selectivity compared to state-of-the-art Pd-based catalyst preparation methods.

## Experimental Section

### Catalyst Preparation

#### Magnetron Sputtering (MS)

All depositions were carried out using a bespoke AJA magnetron sputtering system. The sample was placed into a tailor-made stirring sample holder, stirred the powder during the deposition process, and then loaded into the magnetron sputtering prechamber, reaching 3 × 10^–7^ Torr background pressure in 30 min. The sample holder was transferred to the main chamber, where the background pressure was 3 × 10^–8^ Torr. After closing the gate valve and therefore isolating the main chamber from the prechamber, it took 5 min to stabilize the main chamber background pressure back to 3 × 10^–8^ Torr. The metal atom deposition was carried out with a work pressure of 10 mTorr using Argon, a high-purity Pd target (99.99%) under room temperature. The loading of the magnetron sputtered samples was obtained by inductively coupled plasma-optical emission spectroscopy (ICP-OES) measurements performed on a PerkinElmer Optima 2000 spectrometer.

#### Chemical Vapor Impregnation (CVI)

ZnO was mixed with Pd­(acac)_2_ in appropriate amounts to achieve the desired weight loadings and transferred to a Schlenk flask, which was sealed and lowered into a preheated oil bath at 80 °C. The flask was evacuated under vacuum and heated to 128 °C before being further heated to a temperature of 133 °C at a rate of 1 °C min^–1^, where it was held for 1 h. Following this, the sample was recovered, ground, and calcined in static air (500 °C, 10 °C min^–1^, 16 h) to ensure complete decomposition of the acetylacetonate precursor.

#### Deposition Precipitation (DP)

The appropriate amount of PdCl_2_ was used to prepare a solution (25 mL) in deionized water, and the solution was stirred overnight; 2–4 drops of HCl were added to the solution to ensure the complete dissolution of PdCl_2_. The ZnO support was mixed with deionized water (150 mL) in a 250 mL round-bottomed flask and stirred at 80 °C for 30 min. The metal solution was added to the support slurry and stirred for a further 30 min at 80 °C. Urea (100:1 urea:metal mol mol^–1^) was then added to the mixture and stirred under reflux at 80 °C for 16 h. The catalyst was filtered under vacuum, washed with deionized water (2 L), and dried at 110 °C for 10 h. The catalyst was then recovered and ground to a fine powder.

### Catalyst Testing

The catalyst testing was undertaken using a fixed-bed, continuous flow, 16-bed high-throughput catalytic reactor, which was designed, manufactured, and serviced by Integrated Lab Solutions GmbH (ILS). The reactor was automated using Integrated Workflow Manager software, based on the LabVIEW package, and operated by the Siemens Win CC program. The reactor was divided into 4 heating blocks, each containing 4 catalyst beds. In every reaction, one bed in each block was kept as a blank to ensure comparability. A capillary distribution system coupled with Equilibar back pressure regulators was used to control the gas feed and reactor pressure. A thermocouple was installed in each heating block to control the temperature. The catalyst pellets (0.5 g unless otherwise specified, 425–600 μm pellet size) were mixed with silicon carbide (F80, 190 μm mean particle size) and centered in the isothermal zone of the stainless steel reactor tubes, which had an internal diameter of 4.0 mm. A bed of silicon carbide (F24, 750 μm mean particle size) was used at each end to limit mass transfer, and the reactor tube was plugged with quartz wool. Prior to testing, the catalysts were reduced in situ at 230 or 400 °C for 1 h, with a ramp rate of 5 °C min^–1^, under a flow of 5% H_2_/N_2_ (40 mL min^–1^ flow rate). The reactor temperature was then cooled to 125 °C, and the catalysts were held under N_2_. The system was then pressurized to 20 bar and switched to the reactant gas feed of 20% CO_2_, 60% H_2_, 5% Ar, 15% N_2_ (30 mL min^–1^ flow rate). After a stabilization period of 4 h, the CO_2_ hydrogenation reaction was conducted using the previously reported temperatures of 230–270 °C. Additional experiments were performed in the temperature range of 175–250 °C (Figures S14–S16). A downstream purge of N_2_ (30 mL min^–1^) was used to prevent product buildup, and the downstream oven was held at 120 °C to avoid the condensation of the products. The reaction products were analyzed by online gas chromatography, utilizing an Agilent 7890B system with two flame ionization detectors (FIDs) and one thermal conductivity detector (TCD). The internal standard used was argon. The GC analyzed 4 injections per temperature point per bed via a Vici stream selection valve. CO_2_ was calculated by comparing the moles of CO_2_ in each bed to the moles of CO_2_ in the calibration run at 125 °C. The carbon balance was calculated as the sum of the carbon-containing products (methanol and CO were the only products produced, with small amounts of CH_4_ over certain catalysts) and reactants in the feed divided by the sum of carbon-containing reactants in the calibration runs. Catalyst testing errors were calculated by running 12 commercial Cu/ZnO/Al_2_O_3_ (CZA) standard catalysts using the conditions stated. The error for CO_2_ conversion was ±1% and product selectivity was ± 2%. The error for MeOH productivity was calculated to be a relative standard deviation of ±5%.

### Catalyst Characterization

X-ray photoelectron spectroscopy (XPS) measurements were performed using a Kratos Axis Ultra DLD system using a monochromatic Al Ka X-ray source (photon energy = 1486.6 eV) operating at 144 W (12 mA × 12 kV). Data was collected with pass energies of 160 eV for survey spectra, and 40 eV for the high-resolution scans with step sizes of 1 and 0.1 eV, respectively. The system was operated in Hybrid mode, using a combination of magnetic immersion and electrostatic lenses, and acquired over an area approximately 300 × 700 μm. A magnetically confined charge compensation system using low-energy electrons was used to minimize charging of the sample surface, and all spectra were taken with a 90° take-off angle. A pressure of ca. 5 × 10^–9^ Torr was maintained during the collection of the spectra. For analysis, all samples were pressed onto double-sided adhesive tape attached to a glass microscope slide, itself attached to a Kratos standard sample bar. All data were analyzed using CasaXPS (v2.3.26) after subtraction of a Shirley background and using modified Wagner sensitivity factors as supplied by the instrument manufacturer. Where required, curve fits were performed using a Voigt-type function (LA line shape in CasaXPS) and utilizing line shapes taken from bulk materials. Peak positions were calibrated to the C­(1s) peak of adventitious carbon,^1^ with a secondary reference to the Zn 2p_3/2_ spectra. Experimental error for the peak positions was 0.2 eV. TEM images were acquired at 200 kV accelerating voltage on JEOL 2100F FEG TEM with a Gatan Model 1027 K3-IS direct detection camera (point resolution limit 0.23 nm, lattice resolution limit 0.1 nm).

## Results and Discussion

Pd/ZnO catalysts were synthesized using deposition-precipitation (DP), chemical vapor impregnation (CVI), and magnetron sputtering (MS), and compared against previously reported catalysts from the Hutchings group prepared by incipient wetness impregnation (IM) and sol immobilization (SI). Each catalyst was prepared with a 1 wt % Pd loading on a commercially available ZnO support with a surface area of 8 m^2^ g^–1^ (Figure S1) to ensure consistency across catalyst preparation methods (see Figure 1a, Table S1 and ESI for preparation method details). The activity of the ZnO support without any metal deposition is shown in Table S2. No activity was detected at 230 or 250 °C, with negligible activity observed at 270 °C (ca. 0.2% conversion, 66 mmol_MeOH_ h^–1^ kg^–1^_cat_). For all Pd/ZnO catalysts, the only observed products were methanol and carbon monoxide. The catalysts were evaluated for methanol synthesis from CO_2_ following a prereduction step at 400 °C under H_2_, with reaction conditions set at 250 °C, a CO_2_:H_2_ ratio of 1:3, and a total pressure of 20 bar (Table S3). Remarkably, the MS catalyst outperformed all other catalysts in terms of total CO_2_ conversion and methanol productivity, whether normalized by total catalyst mass or Pd content (Table S3), with additional comparison to literature values provided in Table S4. This result emphasizes the importance of the atomic contact between the Pd catalytic centers and planes of ZnO, necessary for the effective formation of PdZn alloy at the nanoscale. Methanol productivity followed the order of MS > CVI > DP > SI > IM.

**Figure 1:**
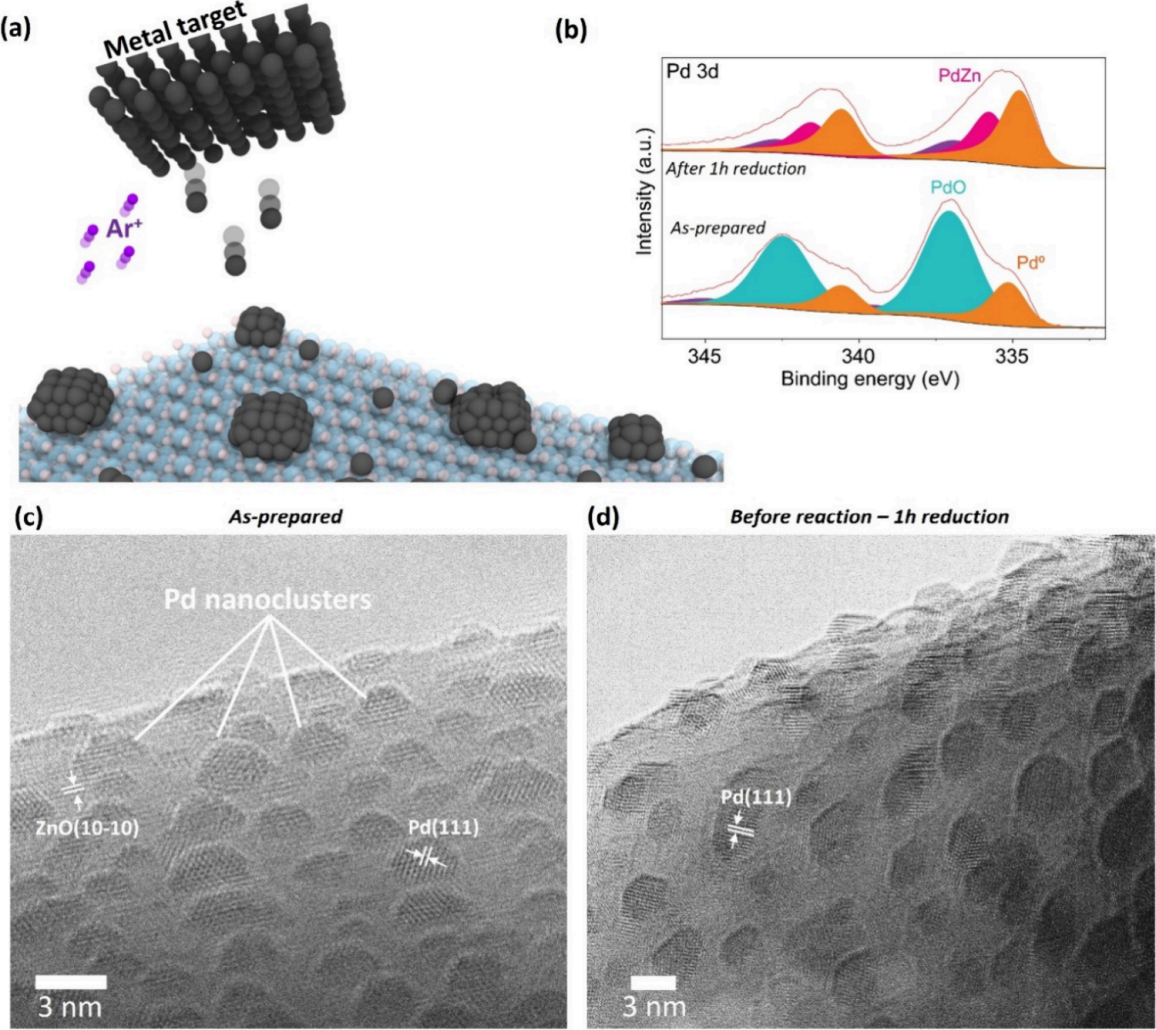
Pd nanoclusters self-assembly on ZnO support using magnetron sputtering. (a) Schematic representation of Pd/ZnO preparation by magnetron sputtering; (b) Pd 3d XPS spectra for Pd/ZnO as-prepared and after a reductive heat treatment, with Pd^0^ shown in orange, PdZn shown in fuschia pink, and PdO shown in turquoise; (c) HR-TEM image showing Pd nanoclusters supported on ZnO; (d) HR-TEM image of Pd/ZnO after a reductive heat treatment at 400 °C to induce the formation of the β-PdZn alloy phase. Lattice fringes observed in (c) and (d) correspond to the (111) fcc lattice planes of Pd metal and hcp lattice planes (10–10) of ZnO.

To link the catalytic performance with the catalyst’s atomic structure, we investigated the structure and chemical environment of the Pd/ZnO catalysts synthesized by MS and CVI before and after the reduction step. HR-TEM images of Pd nanoclusters on ZnO produced by MS show a mean diameter of 2.4 ± 0.5 nm as-prepared, which increases to 3.5 ± 0.7 nm after the reduction step with a very narrow particle size distribution (Figures 1 and S2). In contrast, Pd/ZnO produced by CVI exhibits significantly larger Pd particles, measuring 6.0 ± 2.1 nm after reduction, with a broader size distribution compared to MS (Figure S3). Additionally, for HR-TEM analysis, CO pulse chemisorption measurements were performed in MS and CVI catalysts. For the MS catalyst, CO uptake of 0.34 μmol was measured using 11 mg of catalyst. In comparison, the CVI catalyst exhibited a CO uptake of 0.90 μmol using 61 mg of catalyst. These results show that the MS catalyst has 2.3 times more available sites per gram of catalyst compared to the CVI catalyst. This finding is consistent with the TEM analysis presented in Figures S2b and S3b and further highlights the efficiency of the MS approach in producing high-performing catalysts.

Furthermore, powder X-ray diffraction (XRD) for both catalysts after reduction confirms the β-PdZn alloy phase, indicated by reflections at 41.6 and 44.5°, which are assigned to the (111) and (200) planes, respectively (Figures S10–S12). The more intense reflections observed for the CVI catalyst are indicative of larger PdZn particles compared with the MS catalyst, which is in agreement with the HR-TEM and CO chemisorption data.

X-ray photoelectron spectroscopy (XPS) analysis of the as-prepared MS Pd nanoclusters on ZnO showed the presence of both Pd^0^ and PdO at 335.0 and 337.0 eV, respectively (Figure 1b). Following reduction, a peak assigned to the PdZn alloy was visible at 335.8 eV alongside the metallic Pd^0^ peak.^18,19^ Furthermore, while the Zn Auger spectra were dominated by ZnO at 988.5 eV, the difference spectrum between pure ZnO and Pd/ZnO MS, both reduced at 400 °C, showed the presence of metallic Zn, highlighting the formation of the PdZn alloy (Figure S7). In contrast, no metallic Pd was detected in the CVI catalyst before reduction, with only PdO identified by XPS (Figure S8). After reduction, the XPS spectrum of the CVI catalyst showed the presence of both metallic Pd^0^ and PdZn (Figure S9).^3^

CO–DRIFTS measurements were performed in the 1% Pd/ZnO MS and CVI catalysts to further investigate differences in their electronic environments (Figure S5). The MS catalyst exhibits a peak centered at 2086 cm^–1^, while the CVI catalyst shows a blue-shifted peak at 2093 cm^–1^. In both cases, these signals correspond to linearly bound CO on metallic Pd.^20,21^ Notably, CO can reduce PdO, which explains the appearance of metallic Pd–CO bonding in the CVI catalyst despite its absence in the corresponding XPS spectra.^22^ Additionally, the MS catalyst displays distinct peaks at 1974 and 1924 cm^–1^, attributed to bridge-bonded CO on metallic Pd,^23^ which are not observed for the CVI catalyst. These results indicate stronger backdonation from Pd to CO in the MS catalyst, arising from enhanced charge transfer from Zn to Pd,^24^ facilitated by a pristine and well-defined Pd–ZnO interface. After purging with N_2_ (Figure S5b), the linearly bound CO peak persisted for the 1% CVI catalyst, though at much lower intensity, but was absent for the 1% MS catalyst. This indicates significantly weaker binding of CO on the MS catalyst. Such weaker CO bonding has been linked to enhanced methanol selectivity in PdZn-based systems, as it suppresses CO dissociation to methane and favors methanol formation.^7,25,26^ DRIFTS analysis was performed on both samples after reduction at 400 °C for 1 h. Following CO saturation (Figure S5c), both catalysts exhibit peaks for linearly bound CO around 2090 cm^–1^, along with a broad feature between 1800–2000 cm^–1^ attributed to bridged and triply bonded carbonyl species. A red shift in the linear CO peak is observed for both samples, with a more pronounced shift for the MS catalyst, suggesting the formation of a PdZn alloy. After N_2_ purging, no discernible peaks remain for either sample, indicating weak CO binding to Pd or PdZn.

To summarize, the key difference between the MS and CVI materials lies in the higher fraction of Pd surface atoms in the MS and, critically, the presence of metallic Pd in the MS catalyst prior to reduction, which is absent in the CVI catalyst. This difference directly impacts the alloying process with Zn, as the presence of metallic Pd in the MS promotes alloy formation through a pristine Pd–ZnO interface. In turn, this alters the electronic environment of Pd, affecting its binding strength with both CO and other carbonyl species, as evidenced by DRIFTS analysis, and can ultimately affect the catalyst’s performance in methanol production.^5,25,26^

The detailed catalytic performance of the Pd/ZnO catalysts prepared by MS and CVI is shown in Figure 2 and Table S5. Additionally, to Pd/ZnO catalysts with 1 wt % Pd loading, we prepared catalysts with 0.5, 3, and 5% Pd loading to further investigate the effectiveness of MS as a novel method for catalyst preparation in methanol synthesis. Notably higher CO_2_ conversions and selectivities were achieved for the MS catalysts across all temperatures and weight loadings explored (Figures 2 and S13, Table S5). At reaction temperatures of 230 and 250 °C, the increased conversion corresponded to higher methanol productivity for all MS catalysts as compared with CVI (Figure 2b,c). MS catalysts continue to outperform CVI at 270 °C for the 0.5 and 1 wt % loadings, but for the 3 and 5 wt % catalysts, slightly higher methanol productivity was achieved over the CVI catalysts. This is likely due to thermodynamic equilibrium limitations being approached for the higher-loaded MS catalysts due to their increased conversion.^27^ For instance, for MS catalysts with Pd loadings above 1 wt %, the increase in CO_2_ conversion appears linear rather than exponential (Figure 2a), which deviates from the expected Arrhenius-type kinetic behavior. This deviation, coupled with the sharp drop in methanol productivity at 270 °C (Figure 2b), indicates that these catalysts operate near thermodynamic limitations. It is caused by the fact that Pd/ZnO catalysts are active for both methanol steam reforming and methanol decomposition reactions,^28,29^ both of which may occur under the applied reaction conditions and can reduce methanol yields, particularly for more active catalysts. In contrast, the 0.5 wt % Pd-loaded MS catalyst exhibits an exponential increase in conversion, consistent with kinetic expectations, suggesting that it operates far from equilibrium. This allows it to maintain increasing methanol productivity with the temperature, ultimately surpassing the performance of all CVI catalysts. Therefore, when thermodynamic limitations are mitigated, such as by employing lower Pd loadings, the MS catalysts clearly outperform the CVI catalysts, as evidenced by the comparison between the 0.5 and 5 wt % MS catalysts in Figure 2b. The best yield of methanol achieved for the 3 wt % MS catalyst at 250 °C is 5.0%, which is just 1.5% below the equilibrium yield under these conditions,^30^ and corresponding to 1509 mmol h^–1^ kg^–1^_cat_ (5.4 mol h^–1^ mol^–1^_Pd_). Figure 2c shows methanol productivity normalized to the moles of Pd and highlights the efficient use of MS Pd catalysts with loadings ≤1 wt %. When compared by moles of Pd, the maximum methanol productivity achieved was 16.4 mol h^–1^ mol^–1^_Pd_ for the 0.5 wt % MS catalyst at 270 °C, corresponding to an absolute yield of 2.6%, only 1% below the equilibrium yield of 3.6%.

**Figure 2:**
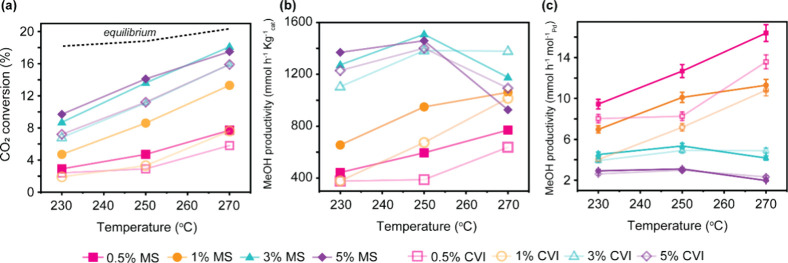
Performance of Pd/ZnO catalysts prepared by MS and CVI. (a) CO_2_ conversion as a function of reaction temperature. Equilibrium data obtained from reference 27 (b) MeOH selectivity vs CO_2_ conversion; (c) MeOH productivity normalized to Pd content as a function of reaction temperature. Catalysts reduced in situ at 400 °C (1 h, 5 °C min^–1^) prior to reaction at 230, 250, and 270 °C with a CO_2_:H_2_ ratio of 1:3 and a total pressure of 20 bar.

Due to the high activity of the 3 and 5 wt % MS catalysts, additional experiments were performed with an increased space velocity (and consequently lower contact time) in the reactor (6000 mL h^–1^ g^–1^_cat_ instead of 3600 mL h^–1^ g^–1^_cat_, Figures S14 and S15) and without the prereduction step. Remarkably, for 5 wt % MS, no difference in performance was observed whether a prereduction step was used or not. The material that was prereduced at 400 °C achieved 13% conversion at 250 °C with 38% methanol selectivity and a methanol productivity of 5.5 mol h^–1^ mol^–1^_Pd_, whereas the catalyst with no prereduction step achieved 13% conversion at 250 °C with 40% methanol selectivity and a methanol productivity of 5.5 mol h^–1^ mol^–1^_Pd_, when a space velocity of 6000 mL h^–1^ g^–1^_cat_ was used. Interestingly, methanol productivity was significantly higher at 6000 than 3600 mL h^–1^ g^–1^_cat_, increasing from 3.1 to 5.5 at 250 °C as the space velocity was increased, with a greater increase in productivity observed upon increasing the space velocity at 270 °C. Similar results were observed for 3 wt % MS upon increasing the space velocity, with CO_2_ conversion decreasing from 14 to 12% and corresponding methanol selectivity increasing from 37 to 44%, resulting in methanol productivities of 5.4 and 9.3 mol h^–1^ mol^–1^_Pd_ for 3600 and 6000 mL h^–1^ g^–1^_cat_, respectively. Both 3 and 5 wt % MS catalysts were active for CO_2_ hydrogenation in the temperature range 175–270 °C, with all data points fitting the previously established linear relationship between methanol selectivity and CO_2_ conversion for PdZn catalysts.^4^ This confirms that a pre-reduction step is not necessary for Pd/ZnO catalysts prepared by MS, highlighting that no further processing steps are required following metal deposition. This further highlights the critical role of metallic Pd before the reduction and reaction steps, as well as the absence of ligands and stabilizers in the catalyst precursor, which can only be provided by the MS method that ensures pure metal in atomic form deposited directly onto the support. It should also be highlighted that no loss of activity or selectivity was observed for any of the Pd/ZnO catalysts over the duration of the testing period (either 96 or 128 h), which shows the high stability of the catalysts prepared by MS (Figure S16). HR-TEM of the MS catalyst after the reaction showed no significant change in particle size compared to that of the catalyst before the reaction, despite the generation of high-temperature steam under the reaction conditions (Figure S2). Similarly, XPS analysis of the postreaction catalyst showed that the PdZn alloy appears stable under the reaction conditions (Figure S6), further highlighting the effectiveness of magnetron sputtering for the preparation of Pd/ZnO catalysts for CO_2_ hydrogenation.

## Conclusions

We have demonstrated an efficient method for producing catalysts by using magnetron sputtering to directly deposit palladium in its atomic form onto a zinc oxide support at room temperature without solvents, reagents, or ligands. This approach allows the formation of Pd nanoclusters with a high fraction of surface atoms and precise control of the metal–support interface, which is crucial for the hydrogenation of CO_2_ to methanol, typically requiring preactivation steps (e.g., interface cleaning through calcination or activation via reduction). By eliminating these steps, we fully utilize the catalyst’s potential from the beginning of the reaction. The productivity of the Pd/ZnO catalyst for CO_2_ conversion to methanol has been shown to be higher than that of catalysts produced using methods like chemical vapor impregnation. This highlights the importance of the metal–support interface for long-term catalyst efficiency. By engineering this interface at the atomic level, we are creating the next generation of heterogeneous catalysts for sustainable chemistry, utilizing rare metals with the greatest atom economy to their fullest potential.

## Supplementary Material



## Notes

Additional experimental details, surface area plot, catalysis results, TEM images, PXRD analysis, CO–DRIFT, and XPS plots (PDF)

## References

[1] Olah G. A. (2005). Beyond
Oil and Gas: The Methanol Economy. Angew. Chem.,
Int. Ed..

[2] Bahruji H., Bowker M., Hutchings G., Dimitratos N., Wells P., Gibson E., Jones W., Brookes C., Morgan D., Lalev G. (2016). Pd/ZnO Catalysts for Direct CO2 Hydrogenation
to Methanol. J. Catal..

[3] Lawes N., Gow I. E., Smith L. R., Aggett K. J., Hayward J. S., Kabalan L., Logsdail A. J., Slater T. J. A., Dearg M., Morgan D. J., Dummer N. F., Taylor S. H., Bowker M., Catlow C. R. A., Hutchings G. J. (2023). Methanol
Synthesis from CO2 and H2
Using Supported Pd Alloy Catalysts. Faraday
Discuss..

[4] Bowker M., Lawes N., Gow I., Hayward J., Esquius J. R., Richards N., Smith L. R., Slater T. J. A., Davies T. E., Dummer N. F., Kabalan L., Logsdail A., Catlow R. C., Taylor S., Hutchings G. J. (2022). The Critical Role of ΒPdZn
Alloy in Pd/ZnO Catalysts for the Hydrogenation of Carbon Dioxide
to Methanol. ACS Catal..

[5] Zabilskiy M., Sushkevich V. L., Newton M. A., Krumeich F., Nachtegaal M., van Bokhoven J. A. (2021). Mechanistic Study of Carbon Dioxide Hydrogenation over
Pd/ZnO-Based Catalysts: The Role of Palladium-Zinc Alloy in Selective
Methanol Synthesis. Angew. Chem., Int. Ed..

[6] Quilis C., Mota N., Pawelec B., Millán E., Navarro Yerga R. M. (2023). Intermetallic PdZn/TiO2 Catalysts for Methanol Production
from CO2 Hydrogenation: The Effect of ZnO Loading on PdZn-ZnO Sites
and Its Influence on Activity. Applied Catalysis
B: Environmental.

[7] Lawes N., Aggett K. J., Smith L. R., Slater T. J. A., Dearg M., Morgan D. J., Dummer N. F., Taylor S. H., Hutchings G. J., Bowker M. (2024). Zn Loading Effects on the Selectivity
of PdZn Catalysts
for CO2 Hydrogenation to Methanol. Catal. Lett..

[8] Bahruji H., Esquius J. R., Bowker M., Hutchings G., Armstrong R. D., Jones W. (2018). Solvent Free Synthesis of PdZn/TiO2
Catalysts for the Hydrogenation of CO2 to Methanol. Top Catal.

[9] Bahruji H., Bowker M., Jones W., Hayward J., Esquius J. R., Morgan D. J., Hutchings G. J. (2017). PdZn Catalysts
for CO2 Hydrogenation
to Methanol Using Chemical Vapour Impregnation (CVI). Faraday Discuss..

[10] Forde M. M., Armstrong R. D., Hammond C., He Q., Jenkins R. L., Kondrat S. A., Dimitratos N., Lopez-Sanchez J. A., Taylor S. H., Willock D., Kiely C. J., Hutchings G. J. (2013). Partial
Oxidation of Ethane to Oxygenates Using Fe- and Cu-Containing ZSM-5. J. Am. Chem. Soc..

[11] Bailey L. A., Douthwaite M., Davies T. E., Morgan D. J., Taylor S. H. (2024). Controlling
Palladium Particle Size and Dispersion as a Function of Loading by
Chemical Vapour Impregnation: An Investigation Using Propane Total
Oxidation as a Model Reaction. Catal. Sci. Technol..

[12] Kohlrausch E. C., Ghaderzadeh S., Aliev G. N., Popov I., Saad F., Alharbi E., Ramasse Q. M., Rance G. A., Danaie M., Thangamuthu M., Young M., Plummer R., Morgan D. J., Theis W., Besley E., Khlobystov A. N., Alves Fernandes J. (2025). One-Size-Fits-All:
A Universal Binding Site for Single-Layer
Metal Cluster Self-Assembly. Adv. Sci..

[13] Popov I., Ghaderzadeh S., Kohlrausch E. C., Norman L. T., Slater T. J. A., Aliev G. N., Alhabeadi H., Kaplan A., Theis W., Khlobystov A. N., Fernandes J. A., Besley E. (2023). Chemical Kinetics of
Metal Single Atom and Nanocluster Formation on Surfaces: An Example
of Pt on Hexagonal Boron Nitride. Nano Lett..

[14] Kohlrausch E. C., Centurion H. A., Lodge R. W., Luo X., Slater T., Santos M. J. L., Ling S., Mastelaro V. R., Cliffe M. J., Goncalves R. V., Fernandes J. A. (2021). A High-Throughput,
Solvent Free Method for Dispersing Metal Atoms Directly onto Supports. J. Mater. Chem. A.

[15] Chen Y., Young B. J., Aliev G. N., Kordatos A., Popov I., Ghaderzadeh S., Liddy T. J., Cull W. J., Kohlrausch E. C., Weilhard A., Hutchings G. J., Besley E., Theis W., Fernandes J. A., Khlobystov A. N. (2025). Evolution of Amorphous Ruthenium
Nanoclusters into Stepped Truncated Nano-Pyramids on Graphitic Surfaces
Boosts Hydrogen Production from Ammonia. Chem.
Sci..

[16] Gonçalves R. V., Vono L. L. R., Wojcieszak R., Dias C. S. B., Wender H., Teixeira-Neto E., Rossi L. M. (2017). Selective Hydrogenation of CO2 into
CO on a Highly Dispersed Nickel Catalyst Obtained by Magnetron Sputtering
Deposition: A Step towards Liquid Fuels. Applied
Catalysis B: Environmental.

[17] Cano I., Weilhard A., Martin C., Pinto J., Lodge R. W., Santos A. R., Rance G. A., Åhlgren E. H., Jónsson E., Yuan J., Li Z. Y., Licence P., Khlobystov A. N., Alves Fernandes J. (2021). Blurring the Boundary between Homogenous
and Heterogeneous Catalysis Using Palladium Nanoclusters with Dynamic
Surfaces. Nat. Commun..

[18] Penner S., Jenewein B., Gabasch H., Klötzer B., Wang D., Knop-Gericke A., Schlögl R., Hayek K. (2006). Growth and Structural Stability of
Well-Ordered PdZn Alloy Nanoparticles. J. Catal..

[19] Holzapfel H. H., Wolfbeisser A., Rameshan C., Weilach C., Rupprechter G. (2014). PdZn Surface
Alloys as Models of Methanol Steam Reforming Catalysts: Molecular
Studies by LEED, XPS. TPD and PM-IRAS. Top Catal.

[20] Tian P., Ouyang L., Xu X., Ao C., Xu X., Si R., Shen X., Lin M., Xu J., Han Y.-F. (2017). The Origin
of Palladium Particle Size Effects in the Direct Synthesis of H2O2:
Is Smaller Better?. J. Catal..

[21] Chen H., Yang B., Zhang Y., Che C., Zhang F., Han W., Wen H., Wang A., Zhang T. (2024). The Geometric and Electronic
Effects of Ceria on Promoting PdZn Catalyst for Enhanced Acetylene
Semi-Hydrogenation. ChemCatChem..

[22] Meng L., Jia A.-P., Lu J.-Q., Luo L.-F., Huang W.-X., Luo M.-F. (2011). Synergetic Effects
of PdO Species on CO Oxidation over
PdO–CeO2 Catalysts. J. Phys. Chem. C.

[23] Hua Z., Wang S., Mu L., Lv S., Xu X., Gu X., Li L. (2024). Carboxyl Pathway-Dominant
HCOOH Dehydrogenation Boosts
the Low Temperature Transfer Hydrogenation Activity of PdZn Catalyst. J. Catal..

[24] Ticali P., Salusso D., Airi A., Morandi S., Borfecchia E., Ramirez A., Cordero-Lanzac T., Gascon J., Olsbye U., Joensen F., Bordiga S. (2023). From Lab to
Technical CO2 Hydrogenation
Catalysts: Understanding PdZn Decomposition. ACS Appl. Mater. Interfaces.

[25] Lawes N., Dummer N. F., Fagan S., Wielgosz O., Gow I. E., Smith L. R., Slater T. J. A., Davies T. E., Aggett K. J., Morgan D. J., Taylor S. H., Hutchings G. J., Bowker M. (2024). CO2 Hydrogenation to Methanol on
Intermetallic PdGa
and PdIn Catalysts and the Effect of Zn Co-Deposition. Applied Catalysis A: General.

[26] Li X., Cheng Q., Zhang Y., Liu Y., Pan Y., Zhao D., Xiong S., Liu W., Jiang X., Yan J., Duan X., Tian Y., Li X. (2025). Engineering Lattice
Dislocations of TiO2 Support of PdZn–ZnO Dual-Site Catalysts
to Boost CO2 Hydrogenation to Methanol. Angew.
Chem., Int. Ed..

[27] Zhong J., Yang X., Wu Z., Liang B., Huang Y., Zhang T. (2020). State of the Art and
Perspectives in Heterogeneous Catalysis of CO2
Hydrogenation to Methanol. Chem. Soc. Rev..

[28] Feng H., Elam J. W., Libera J. A., Setthapun W., Stair P. C. (2010). Palladium Catalysts Synthesized by
Atomic Layer Deposition
for Methanol Decomposition. Chem. Mater..

[29] Iwasa N., Masuda S., Ogawa N., Takezawa N. (1995). Steam Reforming of
Methanol over Pd/ZnO: Effect of the Formation of PdZn Alloys upon
the Reaction. Applied Catalysis A: General.

[30] Shen W.-J., Jun K.-W., Choi H.-S., Lee K.-W. (2000). Thermodynamic Investigation
of Methanol and Dimethyl Ether Synthesis from CO2 Hydrogenation. Korean J. Chem. Eng..

