# Supplementary material for: Direct Formation of the Atomic Pd-ZnO Interface by Magnetron Sputtering Primed for Methanol Production from CO2
Source: ACS Catal. 2025 Aug 22;15(17):15502–8. doi: 10.1021/acscatal.5c04822 (PMC12418300; doi:10.1021/acscatal.5c04822)
Supplement: Supplementary file 1 [file cs5c04822_si_001.pdf]

Supplementary Information

Direct Formation of Atomic Pd-ZnO Interface by Magnetron Sputtering Primed for Methanol Production from CO_2

Louise R. Smith,[a] Emerson C. Kohlrausch,[b] Kieran J. Aggett,[a] Yifan Chen,[b] Isla E. Gow,[a] Andreas Weilhard,[b] Luke T. Norman,[b] Wolfgang Theis,[c] David J. Morgan,[a] Liam Bailey,[a] Andrei N. Khlobystov,[b] Jesum Alves Fernandes,[b] Graham J. Hutchings.[a]*

[a]Max Planck-Cardiff Centre on the Fundamentals of Heterogeneous Catalysis FUNCAT, Translational Research Hub, Cardiff University, Maindy Road, Cardiff CF24 4HQ, UK

[b]School of Chemistry, University of Nottingham, University Park, Nottingham NG7 2RD, UK

[c]Nanoscale Physics Research Laboratory, School of Physics and Astronomy, University of Birmingham, Edgbaston, B15 2TT, UK

*Corresponding author e-mail: hutch@cardiff.ac.uk

Methods

Materials

Zinc oxide (ZnO, >99.99 %) powder, palladium(II) acetylacetonate ($Pd(acac)_2$, > 99.9 %) and palladium(II) chloride ($PdCl_2$, > 99.999 %) were purchased from Merck and used without further purification. The palladium (Pd > 99.95 %) target used for magnetron sputtering was purchased from Kurt J. Lesker. Hydrogen (H_2, 99.999 %, nitrogen (N_2, 99.998 %), carbon dioxide (CO_2, 99.995 %), and argon (Ar, 99.998 %) were all purchased from BOC.

Catalyst characterisation

ICP-OES

The metal loading of the Pd@ZnO catalysts was quantified using inductively coupled plasma–optical emission spectroscopy (ICP-OES) on a PerkinElmer Optima 2000 spectrometer (Table S1). For sample preparation, approximately 10 mg of catalyst powder was subjected to microwave-assisted acid digestion in 2 mL of freshly prepared aqua regia (a 3:1 mixture of hydrochloric and nitric acids). The digestion was carried out at 150 °C for 1 hour to ensure complete dissolution of both palladium and zinc oxide components. After cooling, the digested samples were diluted to a final volume of 10 mL using a 5% (v/v) hydrochloric acid solution to stabilise the metal ions before analysis.

XRD

X-ray diffraction experiments were carried out using a PANalytical X'Pert Pro diffractometer operating at 40 kV, 40 mA using Cu Kα radiation ($\lambda = 1.54 \times 10^{-10}$ m) with a Ge(111) single crystal monochromator. The scanning angle range (2θ) was 10-80 ° and the scan speed was 0.0356 ° s^{-1}. The reflections were referenced against the International Centre for Diffraction Data (ICDD) database to identify phases present.

CO pulse sorption experiments

MS and CVI Pd/ZnO with 1wt% loading were placed in a quartz tube. The samples were heated with a rate of 10 °C min^{-1} to 250°C in 5%H_2 in Ar (30 mL min^{-1}), and then kept for 1 h at 250°C in 5%H_2 in Ar. The H_2 is replaced after 1 h with He and the sample is kept at 250°C for another 2 h under He to ensure the desorption of residual H adatoms. Then, the sample was cooled to 50°C and further purged for 2 h under a feed of 30 mL of He. The small quantities of CO are pulsed to the sample (1.67 µL). Loop temperature was 90°C.

CO-Diffuse Reflectance Infrared Fourier Transform Spectroscopy (DRIFTS)

CO-DRIFTS was performed using a Bruker Invenio fitted with a mercury cadmium telluride (MCT) detector and a Harrick Praying Mantis Reaction cell ((HVC-DRP-4). The reaction cell was initially purged using a flow of N2 before a background spectrum was acquired using KBr.

The sample was then added, and exposed to a flow of 1% CO/N_2 at 20 mL min^{-1} . Measurements were recorded at room temperature every 30 seconds until saturation of the sample had occurred. The feed was then switched to just N2 with measurements recorded until no change was seen in the spectra produced.

Supplementary Data

Table S1. Pd loading obtained by ICP-OMS for all the investigated catalysts.

**Catalyst**	0.5 MS	0.5 CVI	1 MS	1 CVI	1 DP	3 MS	3 CVI	5 MS	5 CVI
**Pd wt%**	0.5 ± 0.2	0.5 ± 0.1	1.0 ± 0.3	1.0 ± 0.1	1.0 ± 0.1	3.0 ± 0.3	3.0 ± 0.1	4.9 ± 0.4	5.0 ± 0.2

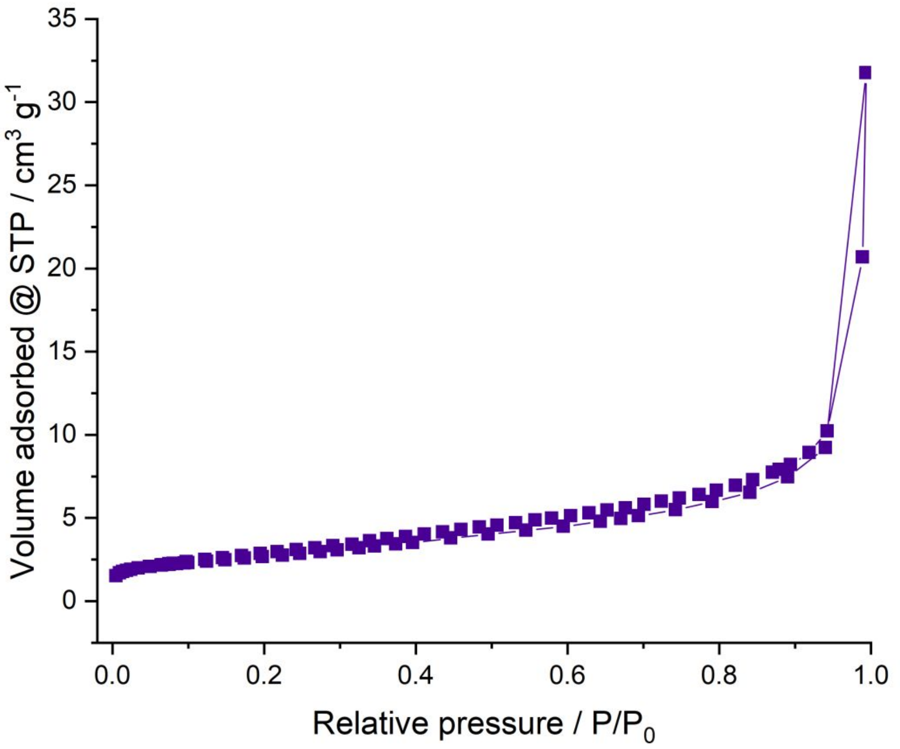

Figure S1. N_2 isotherm of the ZnO support used to calculate surface area in accordance with the B.E.T method.

Table S2. CO_2 conversion, MeOH selectivity and MeOH productivity for bare ZnO after *in situ* reduction at 400 °C

T (°C)	***CO_2 conversion (%)***	***MeOH selectivity (%)***	***MeOH productivity (mmol h^{-1} kg^{-1}cat)***
230	N.D.	N.D.	N.D.
250	N.D.	N.D.	N.D.
270	0.2	74	66

N.D. Not detected. No activity detected at 230 and 250 °C with negligible activity detected at 270 °C.

Table S3. CO_2 conversion, MeOH selectivity and MeOH productivity for 1 wt.% Pd/ZnO catalysts prepared by various techniques and tested at 250 °C with a CO_2:H_2 ratio of 1:3 and a total pressure of 20 bar.

Catalyst	***CO_2 conversion (%)***	***MeOH selectivity (%)***	***MeOH productivity (mol h^{-1} mol^{-1}_{Pd})***	***MeOH productivity (mmol h^{-1} kg^{-1}_{cat})***	***Reference***
1% MS	8.6	36	10.1	949	This work
1% CVI	3.3	52	7.2	674	
1% DP	3.8	46	5.5	520	
1% IM	3.2	22	2.9	270	[2]
1% SI	1.7	76	4.4	410	

DP = deposition precipitation, IM = incipient wetness impregnation, SI = sol immobilisation. All catalysts were prepared using commercially available ZnO from Merck.

Table S4. Comparison of MeOH productivity for various Pd/ZnO based catalysts tested under different reaction conditions.

Catalyst	***MeOH productivity (mmol h^{-1} kg^{-1}_{cat})***	***MeOH productivity (mol h^{-1} mol^{-1}_{Pd})***	***Reaction Conditions***	***Reference***
1% Pd/ZnO (MS)	949	10.1	CO_2:H_2 (1:3) / 20 bar / 250 °C / GHSV of 3600 mL h^{-1} g^{-1}_{cat}.	This work
1% Pd/ZnO (CVI)	674	7.2	CO_2:H_2 (1:3) / 20 bar / 250 °C / GHSV of 3600 mL h^{-1} g^{-1}_{cat}.	
1% Pd/ZnO (DP)	520	5.5	CO_2:H_2 (1:3) / 20 bar / 250 °C / GHSV of 3600 mL h^{-1} g^{-1}_{cat}.	
1% Pd/ZnO (IM)	270	2.9	CO_2:H_2 (1:3) / 20 bar / 250 °C / GHSV of 3600 mL h^{-1} g^{-1}_{cat}.	[2]
1% Pd/ZnO (SI)	410	4.4	CO_2:H_2 (1:3) / 20 bar / 250 °C / GHSV of 3600 mL h^{-1} g^{-1}_{cat}.	
PdZn/Ti-7.5 (4 wt.% Pd)	1797	4.78	CO_2:H_2 (1:3) / 30 bar / 250 °C / GHSV of 12000 mL h^{-1} g^{-1}_{cat}.	[3]
0.5Ca-PdZn/CeO_2 (8.1 wt.% Pd)	3807	5.0	CO_2:H_2 (1:3) / 20 bar / 230 °C / GHSV of 2400 mL h^{-1} g^{-1}_{cat}.	[4]
Pd/a-IGZO (5 wt.% Pd)	2500	5.32	CO_2:H_2 (1:6) / 9 bar / 250 °C / GHSV of 9300 mL h^{-1} g^{-1}_{cat}.	[5]
2-Pd/ZnO-np (2 wt.% Pd)	11985	63.8	CO_2:H_2 (1:3) / 50 bar / 260 °C / GHSV of 120000 mL h^{-1} g^{-1}_{cat}.	[6]

Supplementary note 1

The five first catalysts were tested using the same experimental setup and under identical reaction conditions. The other catalysts were evaluated under a wide range of experimental conditions.

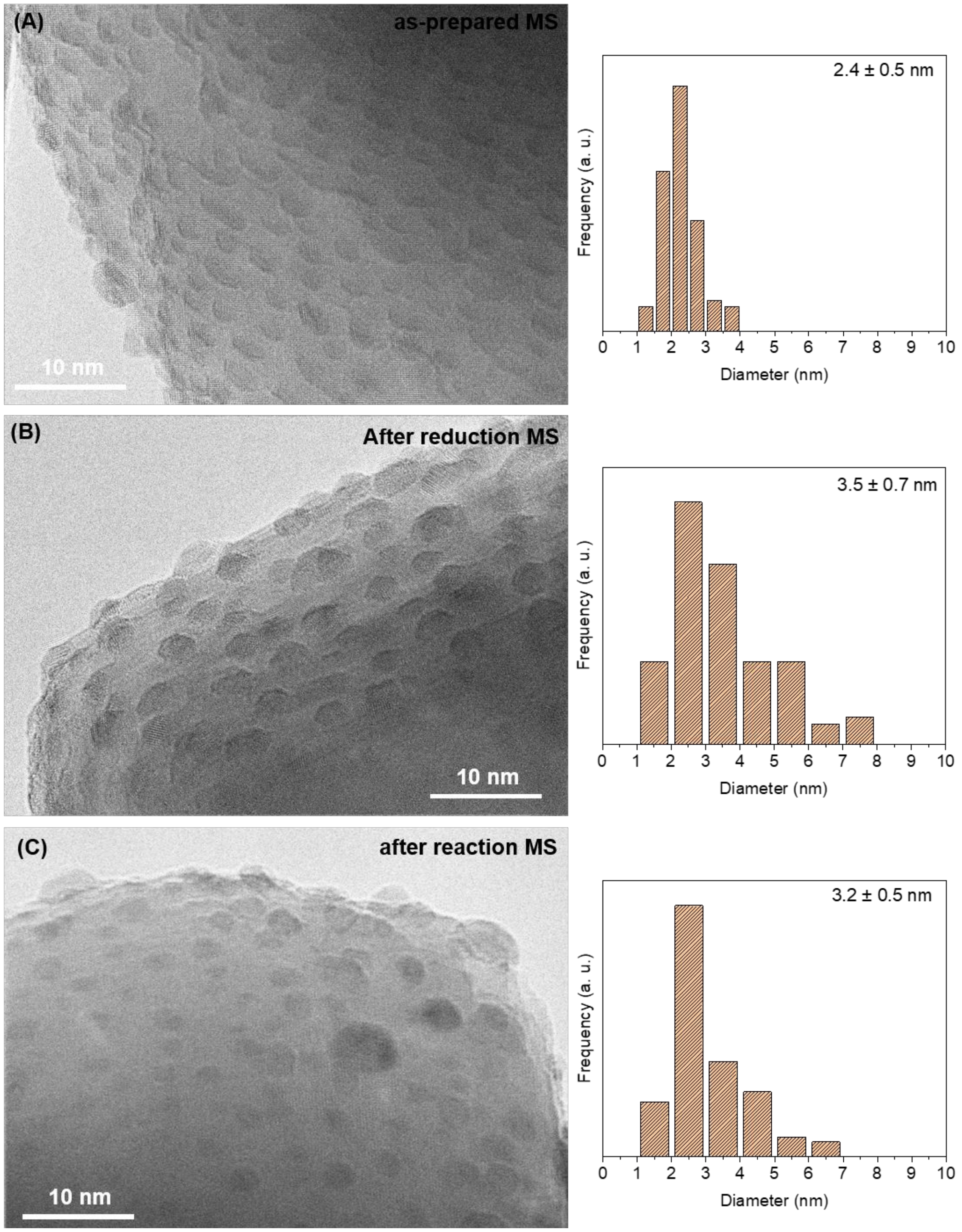

Figure S2. TEM images of MS Pd/ZnO: (A) as-prepared with a mean diameter of 2.4 ± 0.5 nm, (B) after reduction under flowing H_2 for 1 h at 400 °C with a mean diameter of 3.5 ± 0.7 nm, and (C) after reaction with a mean diameter of 3.2 ± 0.5 nm, respectively, with corresponding size distribution diagrams shown beside each HRTEM micrograph.

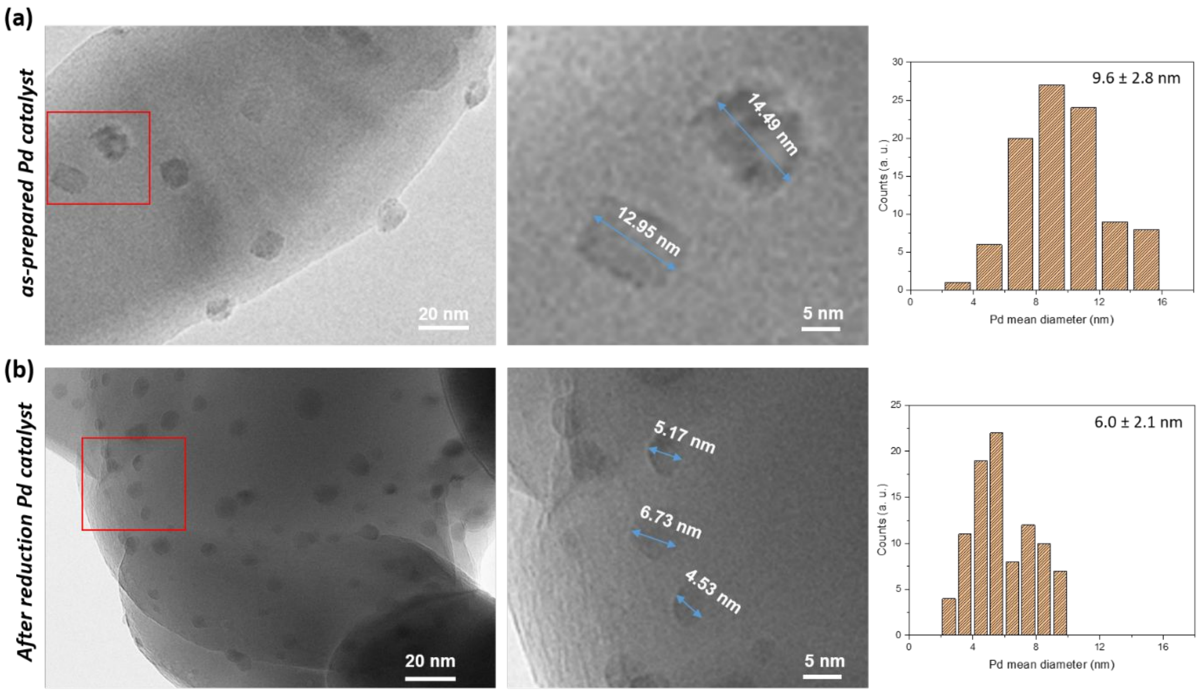

Figure S3. HRTEM images and size distribution histogram of CVI Pd/ZnO: (a) as-prepared and (b) after reduction under flowing H_2 for 1 h at 400 °C, with corresponding size distribution diagrams shown beside each HRTEM micrograph.

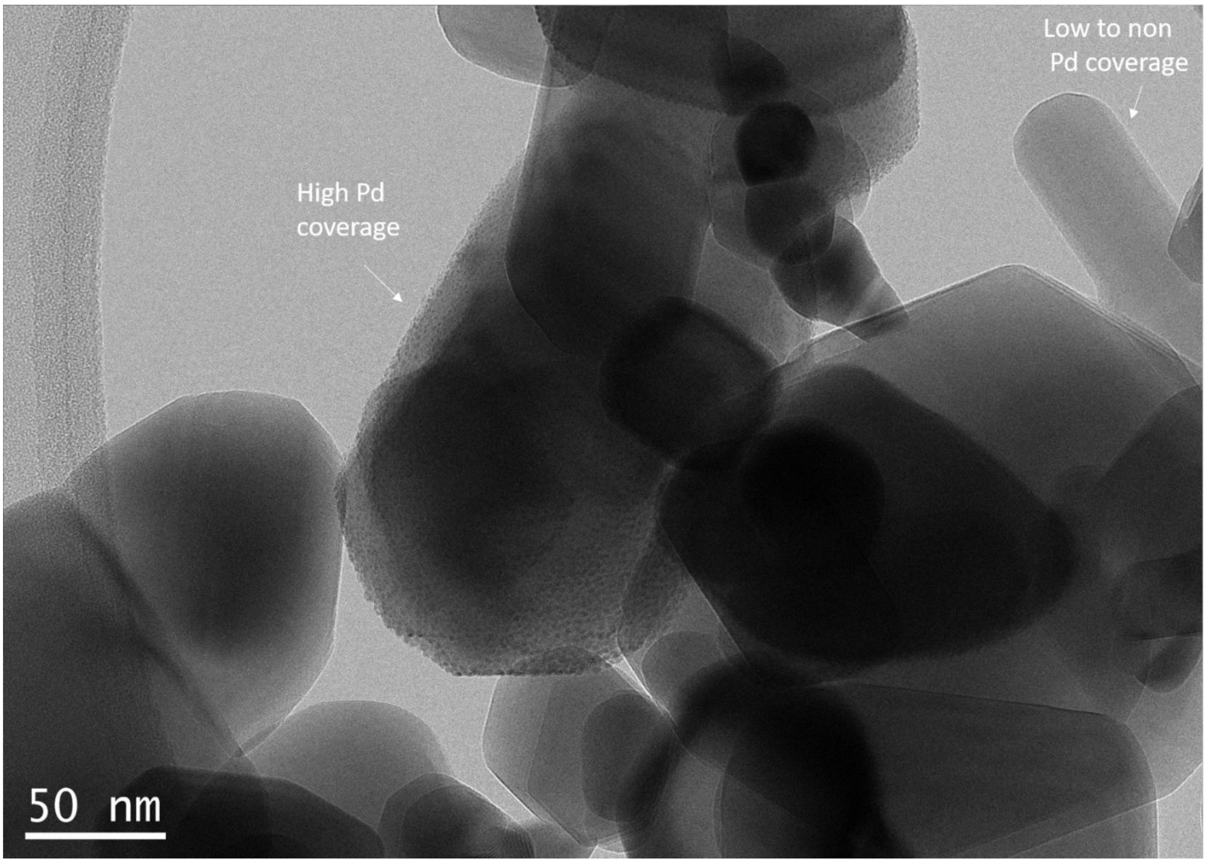

Figure S4. HRTEM image of MS Pd/ZnO showing regions with high Pd particle coverage (>1 wt%) and regions with little to no Pd coverage (<<1 wt%), resulting in an overall average Pd loading of 1 wt% on ZnO. The average Pd loading was measured using ICP-OES, a standard bulk technique for such measurements. Additionally, MS Pd particles are significantly smaller than CVI particles, which also contributes to the difference in Pd coverage. It is important to highlight that the total amount of the Pd-ZnO catalyst system was the same for both CVI and MS in all catalytic experiments.

Supplementary note 2

We have assessed the reproducibility of the MS catalyst by fabricating and testing at least 5–10 independent batches at two different scales (2 g and 10 g). In both cases, the catalytic performance remained consistent. As shown in **Figure 2c**, the variation in methanol productivity between repeated measurements was minimal and comparable to that observed for well-established preparation methods such as CVI. Additionally, new experimental results from CO pulse sorption experiments show that the MS catalyst has 2.3 times more available sites per gram of catalyst compared to the CVI catalyst. This suggests that the MS catalyst does not require a higher loading to achieve the same productivity as catalysts prepared using traditional methods. Therefore, it demonstrates that although there are regions with different coverage, the MS catalyst shows an overall high reproducibility and performance with no significant variation from batch to batch, and even at different production scales.

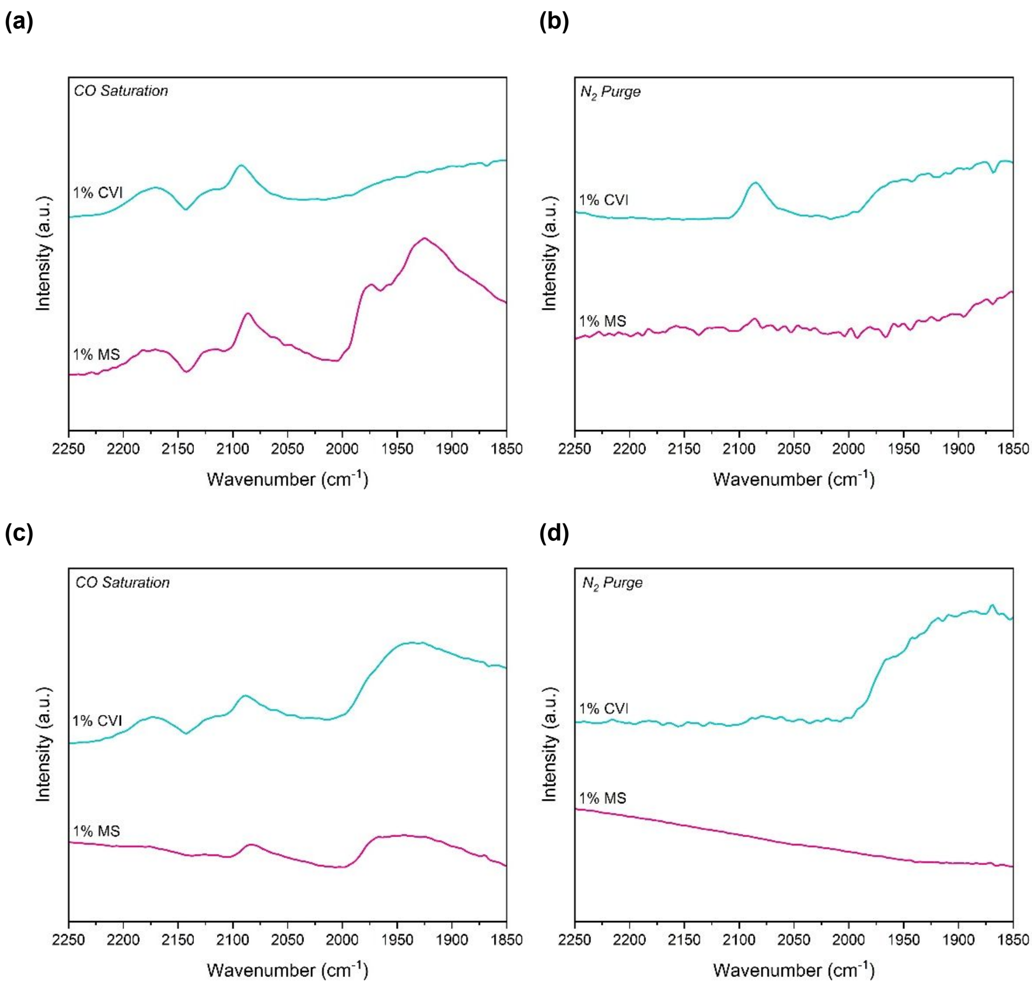

Figure S5. CO-DRIFT spectra of the 1% MS and CVI catalysts; a) & b) as-prepared and c) & d) after reduction at 400°C for 1 hour. a) & c) Spectra taken under flowing CO at saturation level and b) & d) spectra taken under flowing N_2.

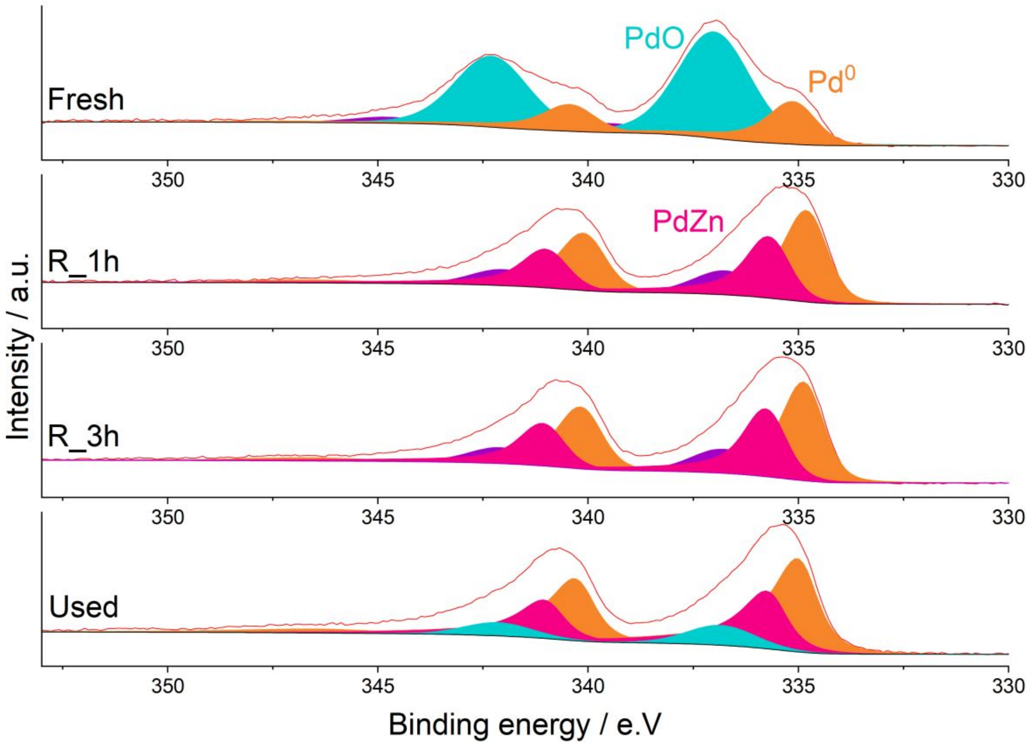

Figure S6. Pd 3d XPS spectra for 1 wt.% Pd/ZnO prepared by MS before and after reduction at 400 °C, and after reaction at 230, 250 and 270 °C with 20 bar pressure.

Supplementary note 3

XPS characterisation of the 1 wt.% Pd/ZnO MS catalyst prior to any reductive heat treatments showed the presence of both Pd^0 (represented by the orange peak at 335.0 eV) and PdO (represented by the blue peak at 337.0 eV), with the latter dominating the spectrum. This was indicative of surface oxidation having occurred following preparation of the material. Following reduction under flowing H_2 at 400 °C for 1 hour, the peak assigned to PdO was no longer visible, suggesting complete surface reduction of Pd occurred. In addition to the Pd^0 peak, a peak assigned to PdZn alloy (represented by the pink peak at 335.8 eV) was also present, in agreement with the presence of the β-PdZn observed by XRD (figure S2). Increasing the time of the reductive heat treatment from 1 hour to 3 hours led to a very minor increase in the ratio of PdZn alloy peak area relative to the Pd^0 peak. A third minor species was also observed (shown by the purple peak at 337.4 eV) which was attributed to Pd^{2+} species in the form of $PdCl_2$. Following reaction, the used catalyst showed the presence of both Pd^0 and PdZn, similarly to the reduced sample, along with a small PdO contribution. The latter was attributed to the formation of water in CO_2 hydrogenation, and the exposure of the sample to air following the reaction and prior to XPS analysis.

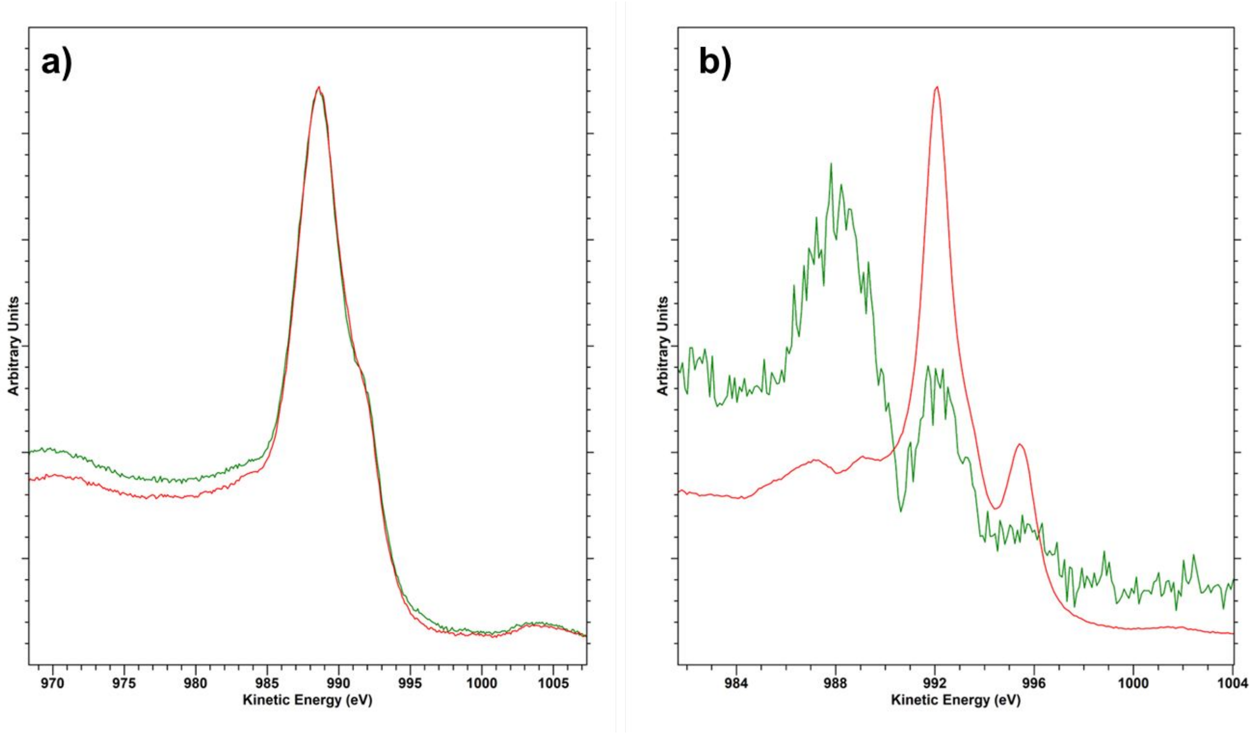

Figure S7. Zn Auger spectra. a) Overlay of Zn Auger for pure ZnO (red) and MS R_3h (green), note the intensity difference around 996 eV; b) Overlap of Zn Auger for metallic Zn (red) and a difference spectrum between the two spectra in a) (green). Note the similar positions of the peaks highlighting the formation of metallic/alloyed Zn.

Supplementary note 4

Both the Zn 2p and Zn Auger spectra were dominated by the presence of ZnO at 988.5 eV for all samples. Nevertheless, following reduction at 400 °C, the difference spectra between pure ZnO and MS R_3h showed the presence of metallic Zn in the reduced Pd/ZnO sample prepared by MS, providing further confirmation to the formation of the PdZn alloy.

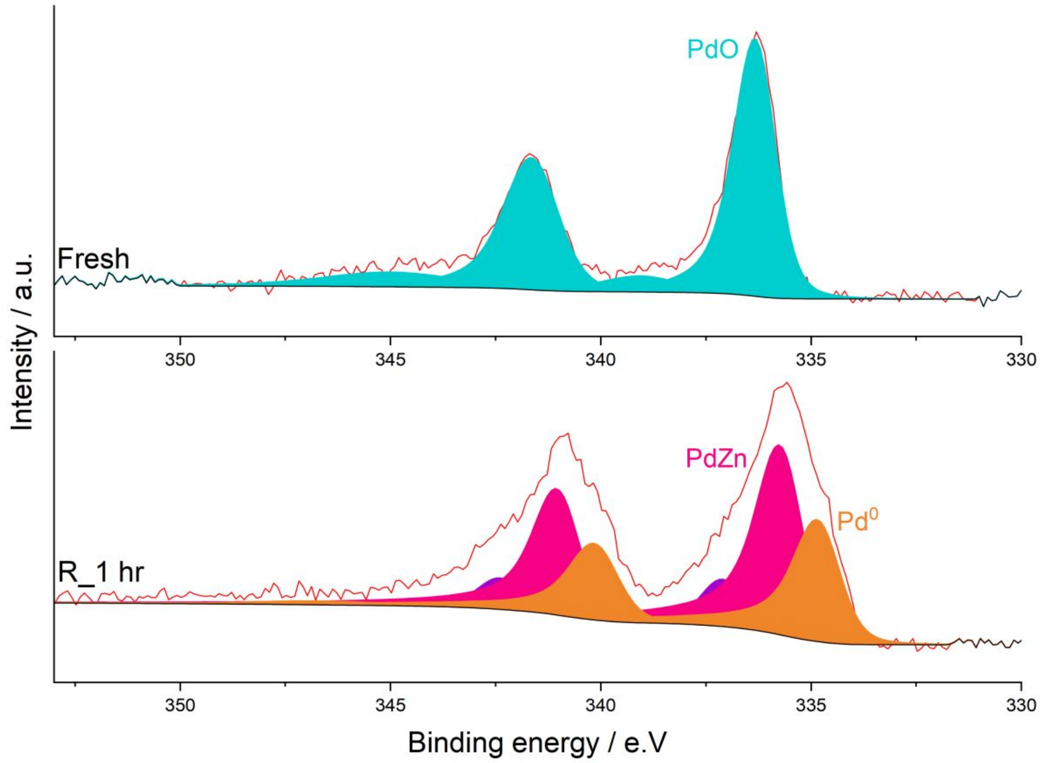

Figure S8. Pd 3d XPS spectra for 1 wt.% Pd/ZnO prepared by CVI before and after reduction at 400 °C.

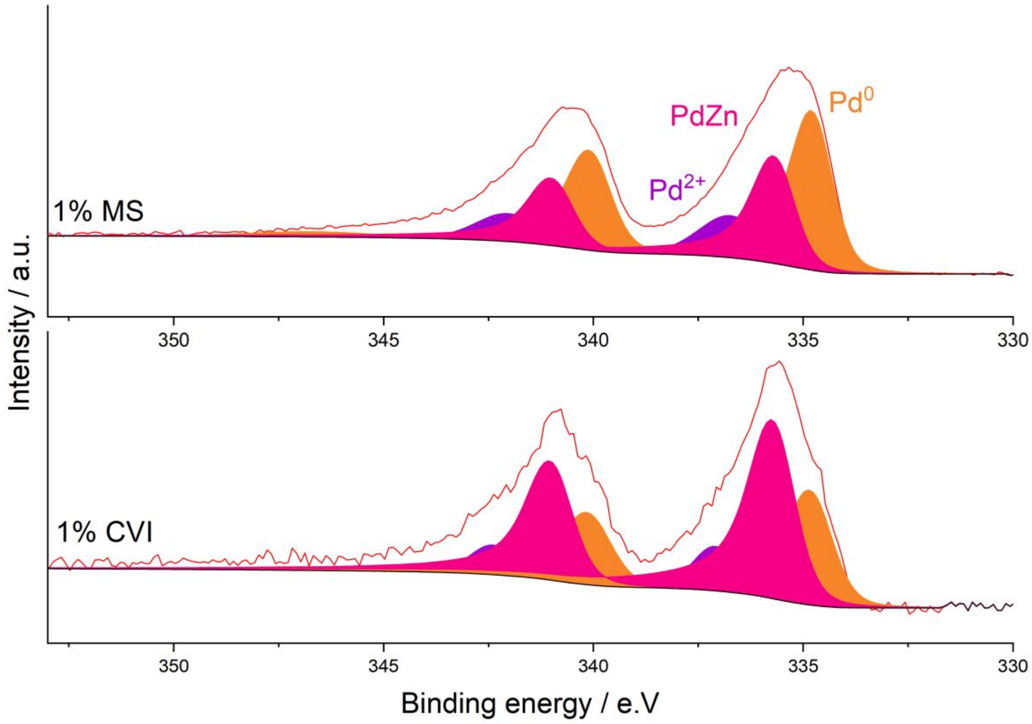

Figure S9. Pd 3d XPS spectra for 1 wt.% Pd/ZnO prepared by MS and CVI after reduction at 400 °C for 1 hour.

Supplementary note 5

Following reduction at 400 °C for 1 hour, similar Pd spectra were obtained for 1 wt.% Pd catalysts prepared by MS and CVI, with both showing the presence of Pd^0 and PdZn. Despite both catalysts having comparable Pd loadings, XPS shows a higher atomic concentration of Pd relative to ZnO for the MS material (3.8 %) compared with the CVI material (1.6 %), indicative of higher Pd dispersion and smaller particle size in the MS catalyst.

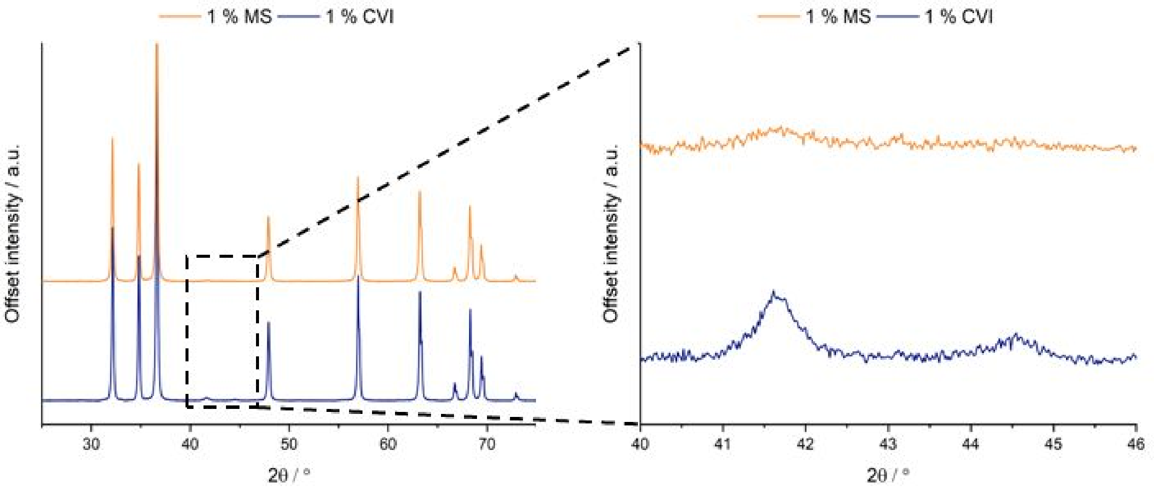

Figure S10. XRD patterns for Pd/ZnO catalysts prepared by MS and CVI after reduction at 400 °C. Diffraction pattern shown between 40 – 46° to highlight the PdZn reflections at 41.6 and 44.5°.

Supplementary note 6

The formation of the *β*-PdZn alloy phase is well established for Pd/ZnO catalysts prepared by CVI through thermal treatments under a reductive atmosphere.[7–9] XRD diffraction patterns for 1 wt.% Pd/ZnO catalysts prepared by both MS and CVI showed the presence of the *β*-PdZn alloy phase, represented by the reflections observed at 41.6 and 44.5°. Whilst comparison of the PdZn crystallite sizes using the Scherrer equation was not possible due to the broadness and weak intensity of the PdZn reflection in the MS catalyst, the sharper peaks visible in the diffraction pattern for the CVI catalyst are indicative of larger PdZn crystallites.

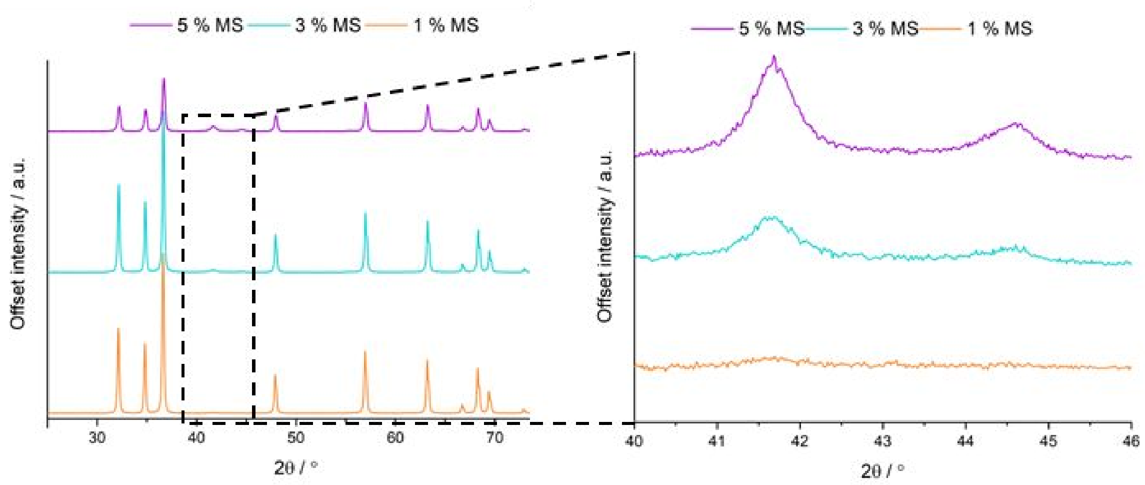

Figure S11. XRD patterns for Pd/ZnO catalysts with a range of Pd loadings prepared by MS after reduction at 400 °C. Diffraction pattern shown between 40 – 46° to highlight the PdZn reflections at 41.6 and 44.5°.

Supplementary note 7

Following reduction at 400 °C (5 °C min^{-1}, 1 hour, 5 % H_2/Ar), XRD patterns for Pd/ZnO catalysts prepared by MS show the presence of the β-PdZn alloy phase indicated by the reflections at 41.6 and 44.5°, assigned to the (111) and (200) planes, respectively (ICSD Collection Code 180143). All other reflections were indexed to the hexagonal wurtzite ZnO phase (ICSD Collection Code 26170). No PdZn reflections were observed for the 0.5 wt% catalyst, and the intensity of the PdZn reflections increased with increasing Pd loading, indicating an increase in PdZn crystallite size with higher metal loadings. Scherrer analysis of the PdZn (111) reflection gave crystallite sizes of 11 and 15 nm for the 3 and 5 wt.% catalysts, respectively. It was not possible to apply to the Scherrer equation to the 1 wt.% material due to the low intensity and broadness of the PdZn reflections.

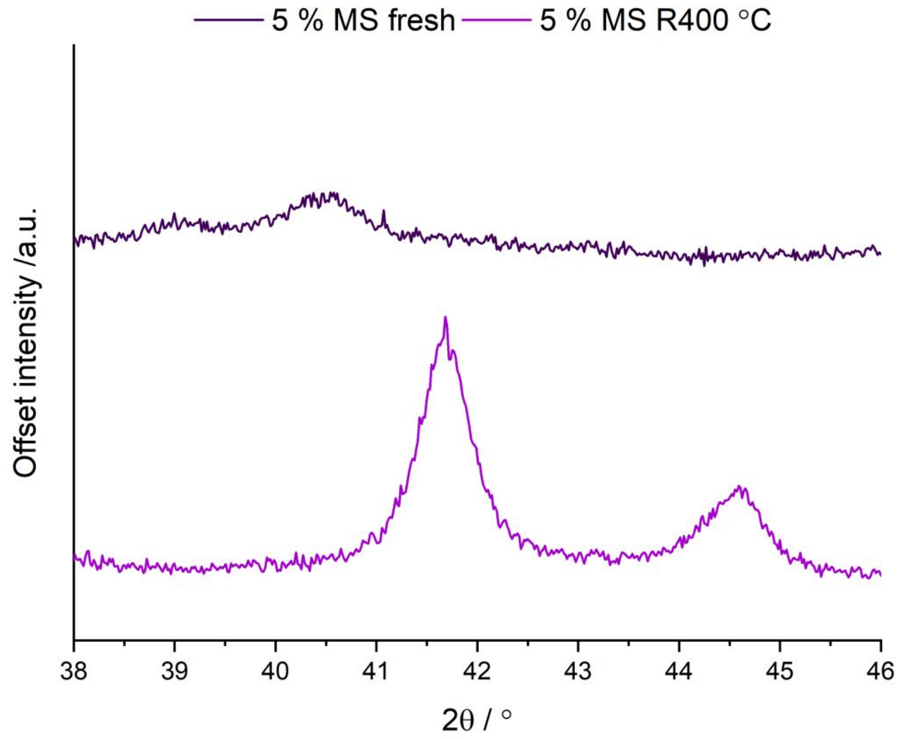

Figure S12. XRD patterns for 5 % Pd/ZnO catalysts prepared by MS before and after reduction at 400 °C. Diffraction pattern shown between 40 – 46° to highlight the PdZn reflections at 41.6 and 44.5° and the Pd reflection at 40.4°.

Supplementary note 8

Due to the increased intensity of the PdZn reflections for the 5 wt.% MS catalyst after reduction at 400 °C compared with the materials with lower Pd loadings, the XRD pattern of the as-prepared material was also obtained. A weak reflection was observed at 2θ = 40.4° which was indexed to the (111) plane of Pd (ICSD Collection Code 76148), highlighting the deposition as metallic Pd in MS catalysts. No reflections other than ZnO were observed for the materials with less than 5 wt.% Pd.

.

Table S5. MeOH productivity, CO_2 conversion and MeOH selectivity for Pd/ZnO catalysts prepared by MS and CVI. Catalysts reduced *in situ* at 400 °C (1 hour, 5 °C min^{-1}) prior to reaction at 230, 250 and 270 °C with a CO_2:H_2 ratio of 1:3, a total pressure of 20 bar and a GHSV of 3600 mL h^{-1} g^{-1}_{cat}.

Catalyst	***MeOH productivity (mol h^{-1} mol^{-1}_{Pd})***			***MeOH productivity (mmol h^{-1} kg^{-1}cat)***			***CO_2 conversion (%)***			***MeOH selectivity (%)***		
T (°C)	*230*	*250*	*270*	*230*	*250*	*270*	*230*	*250*	*270*	*230*	*250*	*270*
0.5 MS	9.5	12.7	16.4	444	595	771	2.9	4.7	7.7	52	42	34
0.5 CVI	8.0	8.3	13.6	377	388	638	2.4	2.9	5.8	51	42	36
1 MS	7.0	10.1	11.3	655	949	1061	4.7	8.6	13.3	46	36	26
1 CVI	4.0	7.2	10.8	378	674	1015	1.9	3.3	7.6	63	52	41
3 MS	4.5	5.4	4.2	1273	1509	1176	8.7	13.6	18.1	48	37	22
3 CVI	3.9	4.9	4.9	1105	1383	1378	6.8	11.1	15.9	53	41	28
5 MS	2.9	3.1	2.0	1369	1459	927	9.7	14.1	17.5	46	34	18
5 CVI	2.6	2.9	2.3	1230	1402	1093	7.2	11.2	15.9	54	39	22

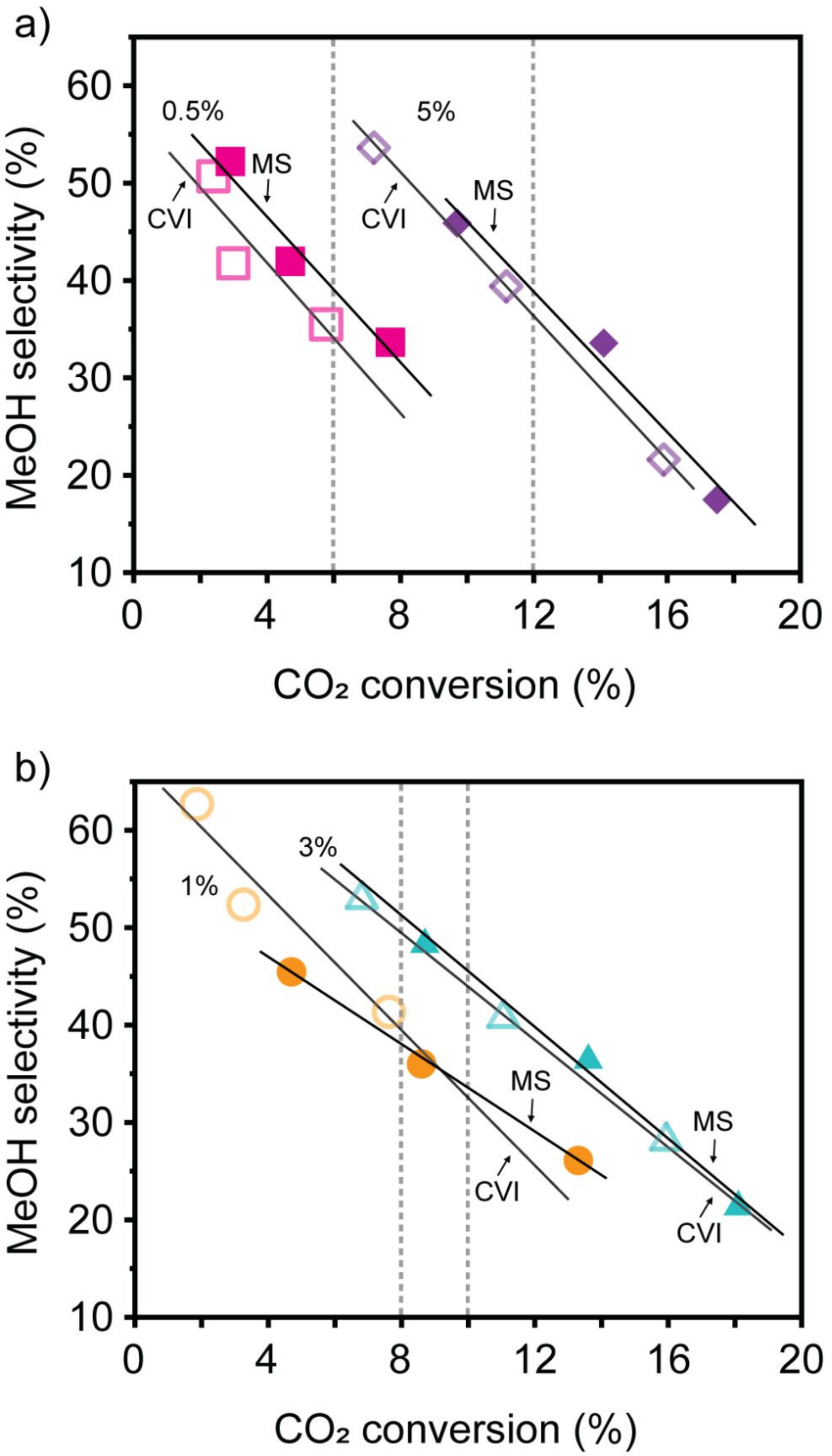

Figure S13. MeOH selectivity as a function of CO_2 conversion for Pd/ZnO catalysts with a range of loadings prepared by MS and CVI. a) Pd/ZnO with 0.5 wt% and 5 %wt Pd loadings, and b) 1 wt% and 3 wt% Pd loadings. Catalysts reduced *in situ* at 400 °C (1 hour, 5 °C min^{-1}) prior to reaction at 230, 250 and 270 °C with a CO_2:H_2 ratio of 1:3, a total pressure of 20 bar and a GHSV of 3600 mL h^{-1} g^{-1}_{cat}. The dotted lines indicate that, at the same conversion, the MS catalyst exhibits higher selectivity than CVI across the loadings used in this study.

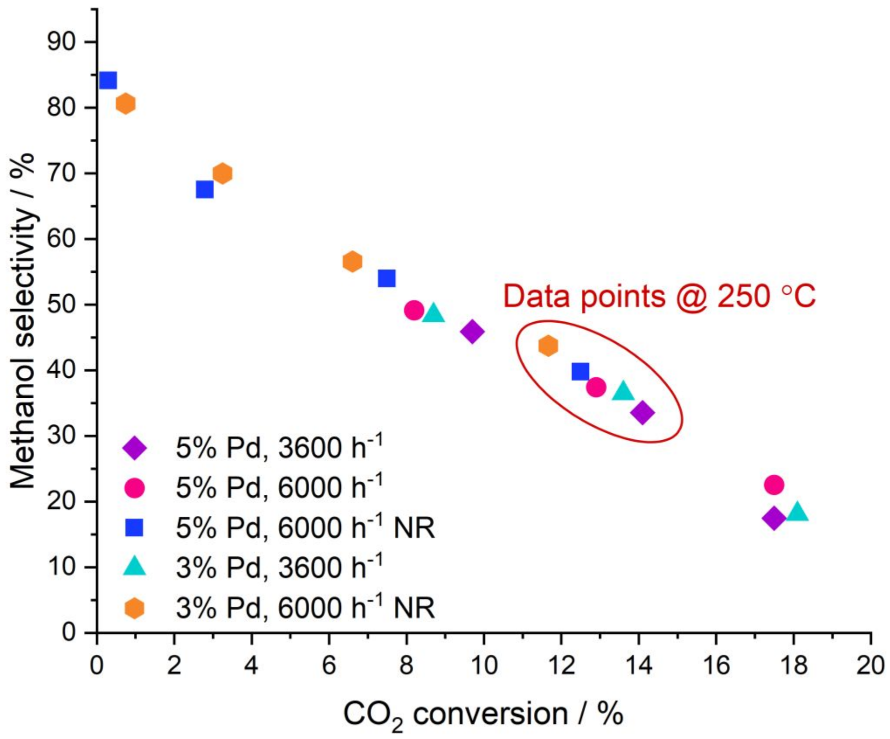

Figure S14. Methanol selectivity *vs.* CO_2 conversion for a range of Pd/ZnO catalysts prepared by MS. Catalysts are reduced *in situ* at 400 °C (5 °C min^{-1}, 1 h) with the exception of catalysts denoted by NR which have no pre-reduction step. The GHSV (mL h^{-1} g^{-1}_{cat}) used is indicated in the legend. Catalysts with a reduction step were reacted at 230, 250 and 270 °C with a CO_2:H_2 ratio of 1:3 and a total pressure of 20 bar. Catalysts without a reduction step were reacted at 175, 200, 225 and 250 °C. Data points obtained at 250 °C are highlighted.

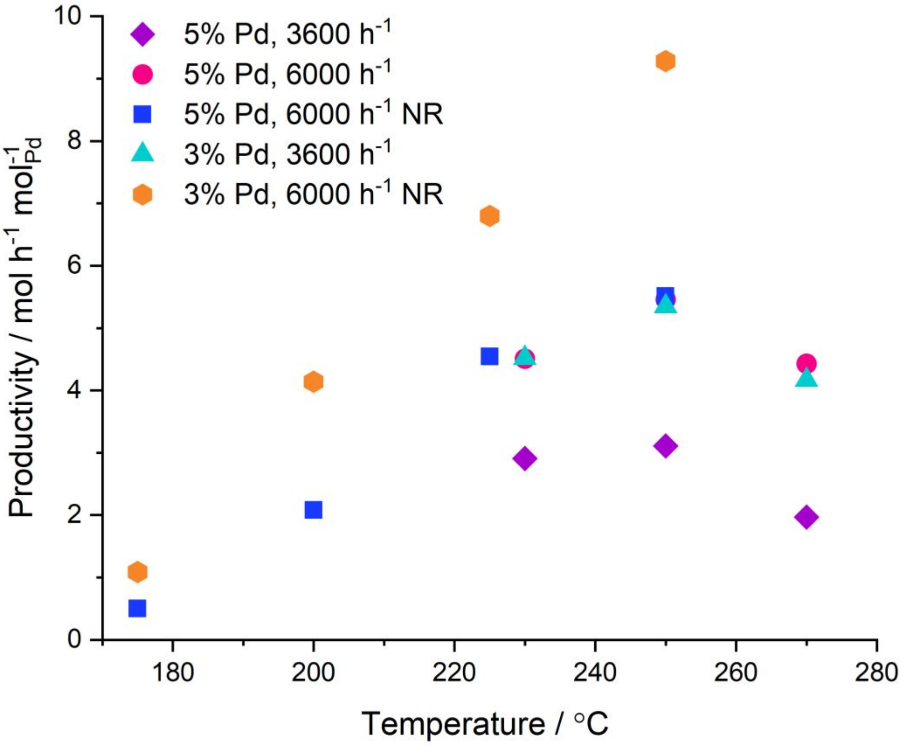

Figure S15. Methanol productivity at different temperatures for a range of Pd/ZnO catalysts prepared by MS. Catalysts are reduced *in situ* at 400 °C (5 °C min^{-1}, 1 h) with the exception of catalysts denoted by NR which have no pre-reduction step. The GHSV (mL h^{-1} g^{-1}_{cat}) used is indicated in the legend. Catalysts were reacted at 175, 200, 225 and 250 °C or 230, 250 and 270 °C with a CO_2:H_2 ratio of 1:3 and a total pressure of 20 bar.

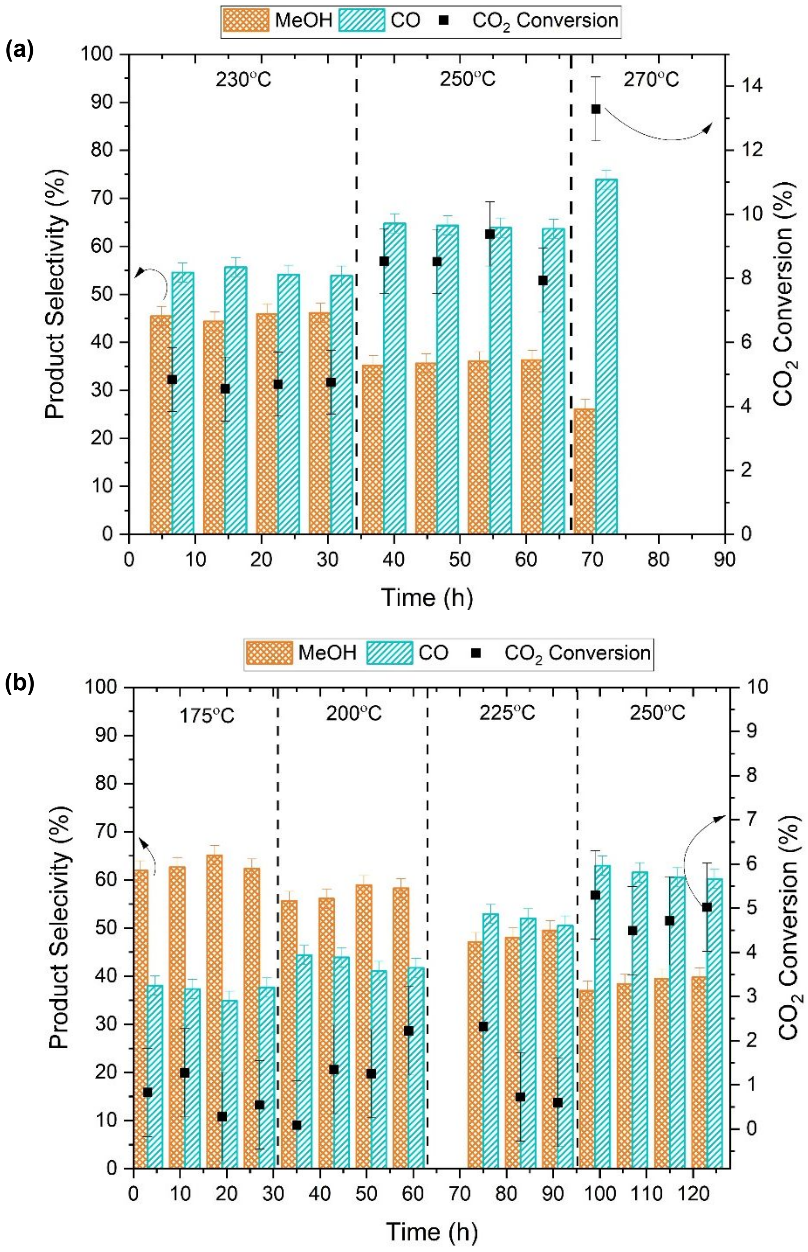

Figure S16. Product selectivity and CO_2 conversion plots for the 1% MS Pd/ZnO catalyst under different reaction conditions. Conditions: a) 96 hrs on-line, 230, 250 and 270 °C with a CO_2:H_2 ratio of 1:3, a total pressure of 20 bar and a GHSV of 3600 mL h^{-1} g^{-1}_{cat} & b) 128hrs on-line, 175, 200, 225 and 250 °C with a CO_2:H_2 ratio of 1:3 and a total pressure of 20 bar and a GHSV of 6000 mL h^{-1} g^{-1}_{cat}.

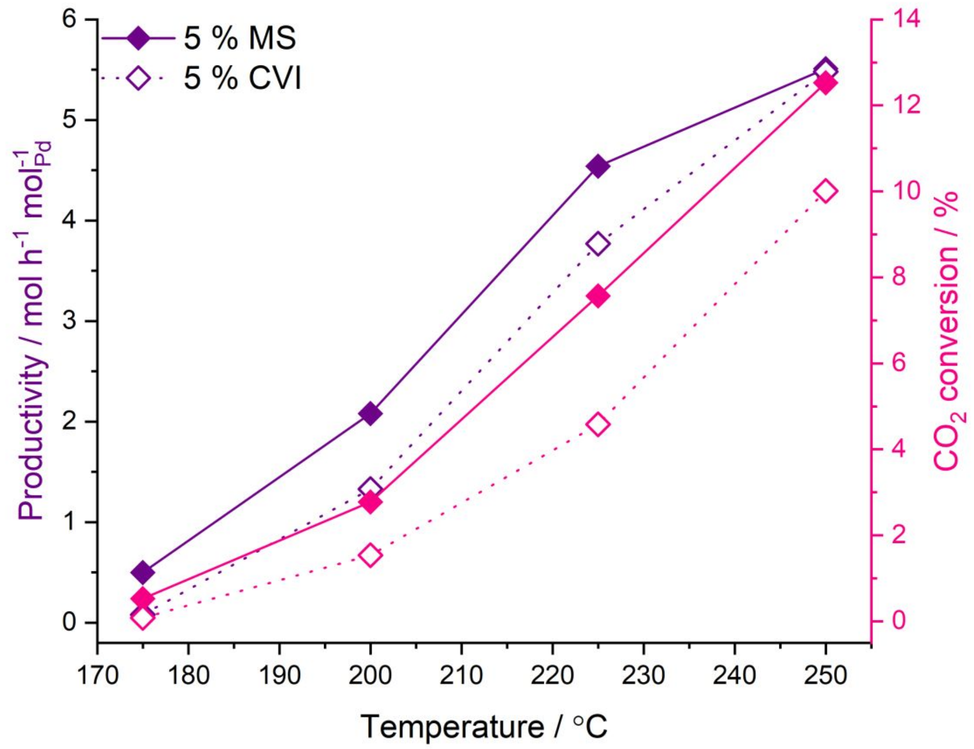

Figure S17. Methanol productivity and CO_2 conversion over 5% Pd/ZnO catalysts (6000 mL h^{-1} g^{-1}_{cat}) prepared by MS (solid symbols) and CVI (hollow symbols) and tested at a temperature range of 175 – 250 °C with a CO_2:H_2 ratio of 1:3 and a total pressure of 20 bar. No pre-reduction step was used.

Supplementary note 9

Similarly to the higher reaction temperature range shown in figure 1, notably higher CO_2 conversions were observed for the 5 wt.% Pd/ZnO catalyst prepared by MS, compared with the CVI analogue, when tested between 175-250 °C. This corresponded to higher methanol productivities over the MS catalyst (with the exception of 250 °C where no significant difference in methanol productivity was observed between the catalysts), highlighting the superior activity of the MS catalyst across a range of reaction temperatures.

References

(1) Davies, P. R.; Morgan, D. J. Practical Guide for X-Ray Photoelectron Spectroscopy: Applications to the Study of Catalysts. *Journal of Vacuum Science & Technology A* **2020**, *38* (3), 033204. https://doi.org/10.1116/1.5140747.

(2) Bahruji, H.; Bowker, M.; Hutchings, G.; Dimitratos, N.; Wells, P.; Gibson, E.; Jones, W.; Brookes, C.; Morgan, D.; Lalev, G. Pd/ZnO Catalysts for Direct CO2 Hydrogenation to Methanol. *Journal of Catalysis* **2016**, *343*, 133–146. https://doi.org/10.1016/j.jcat.2016.03.017.

(3) Quilis, C.; Mota, N.; Pawelec, B.; Millán, E.; Navarro Yerga, R. M. Intermetallic PdZn/TiO2 Catalysts for Methanol Production from CO2 Hydrogenation: The Effect of ZnO Loading on PdZn-ZnO Sites and Its Influence on Activity. *Applied Catalysis B: Environmental* **2023**, *321*, 122064. https://doi.org/10.1016/j.apcatb.2022.122064.

(4) Zaman, S. F.; Ojelade, O. A.; Alhumade, H.; Mazumder, J.; Mohamed, H. O.; Castaño, P. Elucidating the Promoting Role of Ca on PdZn/CeO2 Catalyst for CO2 Valorization to Methanol. *Fuel* **2023**, *343*, 127927. https://doi.org/10.1016/j.fuel.2023.127927.

(5) Fukumoto, K.; Tsuji, H.; Tsuji, M.; Koike, M.; Takatani, K.; Shimizu, M.; Kitano, M.; Hosono, H. CO2 Conversion to Methanol by Hydrogen Species on N-Type Oxide Semiconductors. *J. Am. Chem. Soc.* **2025**, *147* (26), 22634–22641. https://doi.org/10.1021/jacs.5c03910.

(6) Zabilskiy, M.; Sushkevich, V. L.; Newton, M. A.; Krumeich, F.; Nachtegaal, M.; van Bokhoven, J. A. Mechanistic Study of Carbon Dioxide Hydrogenation over Pd/ZnO-Based Catalysts: The Role of Palladium–Zinc Alloy in Selective Methanol Synthesis. *Angewandte Chemie International Edition* **2021**, *60* (31), 17053–17059. https://doi.org/10.1002/anie.202103087.

(7) Bahruji, H.; Bowker, M.; Jones, W.; Hayward, J.; Esquius, J. R.; Morgan, D. J.; Hutchings, G. J. PdZn Catalysts for CO2 Hydrogenation to Methanol Using Chemical Vapour Impregnation (CVI). *Faraday Discuss.* **2017**, *197* (0), 309–324. https://doi.org/10.1039/C6FD00189K.

(8) Bowker, M.; Lawes, N.; Gow, I.; Hayward, J.; Esquius, J. R.; Richards, N.; Smith, L. R.; Slater, T. J. A.; Davies, T. E.; Dummer, N. F.; Kabalan, L.; Logsdail, A.; Catlow, R. C.; Taylor, S.; Hutchings, G. J. The Critical Role of BPdZn Alloy in Pd/ZnO Catalysts for the Hydrogenation of Carbon Dioxide to Methanol. *ACS Catal.* **2022**, *12* (9), 5371–5379. https://doi.org/10.1021/acscatal.2c00552.

(9) Lawes, N.; Gow, I. E.; Smith, L. R.; Aggett, K. J.; Hayward, J. S.; Kabalan, L.; Logsdail, A. J.; Slater, T. J. A.; Dearg, M.; Morgan, D. J.; Dummer, N. F.; Taylor, S. H.; Bowker, M.; Catlow, C. R. A.; Hutchings, G. J. Methanol Synthesis from CO2 and H2 Using Supported Pd Alloy Catalysts. *Faraday Discuss.* **2023**, *242* (0), 193–211. https://doi.org/10.1039/D2FD00119E.
